# Marine Os isotopic evidence for multiple volcanic episodes during Cretaceous Oceanic Anoxic Event 1b

**DOI:** 10.1038/s41598-020-69505-x

**Published:** 2020-07-28

**Authors:** Hironao Matsumoto, Junichiro Kuroda, Rodolfo Coccioni, Fabrizio Frontalini, Saburo Sakai, Nanako O. Ogawa, Naohiko Ohkouchi

**Affiliations:** 1 Atmosphere and Ocean Research Institute, The University of Tokyo, Tokyo, Japan; 2 DiSPeA, University of Urbino, Urbino, Italy; 3 Japan Agency for Marine-Earth Science and Technology, Kanagawa, Japan

**Keywords:** Environmental sciences, Ocean sciences

## Abstract

The Aptian–Albian boundary is marked by one of the major oceanic perturbations during the Cretaceous, called Oceanic Anoxic Event (OAE) 1b. Extensive volcanic episodes at the Southern Kerguelen Plateau has been suggested as the trigger of OAE1b, but compelling evidence remains lacking. Here, we reconstructed the temporal variations of marine Os isotopic ratios across the Aptian–Albian boundary in the Tethyan and Pacific pelagic sedimentary records to elucidate the causal links between OAE1b, the biotic turnover, and volcanic episodes. Our new Os isotopic records show two negative spikes that correlate with a period of planktonic foraminiferal turnover across the Aptian–Albian boundary during OAE1b and suggest multiple submarine volcanic events. By comparing our Os isotopic profile with carbon isotopic compositions of carbonate, CaCO_3_ content, and the relative abundances of agglutinated foraminifera, we conclude that ocean acidification caused by the massive release of CO_2_ through extensive volcanic episodes could have promoted the major planktonic foraminiferal turnover during OAE1b.

## Introduction

**Figure 1:**
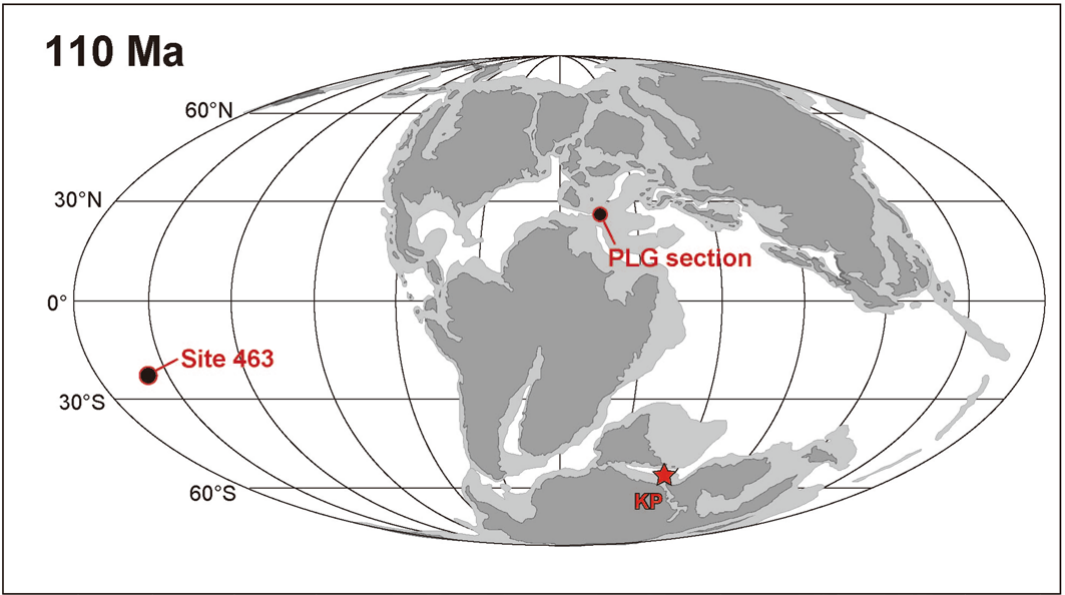
Palaeogeographical reconstruction at 110 Ma based on ref^5^. Red circles represent the locations of the PLG section and DSDP Site 463. The red star indicates the Southern Kerguelen Plateau (KP). The map was created using Illustrator CS5.5 (https://www.adobe.com/products/illustrator.html).

The mid-Cretaceous (Barremian–Turonian) is punctuated by repeated Oceanic Anoxic Events (OAEs), which represent intervals of global and episodic burial of organic-rich sediments on the seafloor under oxygen-deficient bottom waters. Several organic-rich sedimentary deposits are recorded in the uppermost Aptian to the Lower Albian, mainly in the Tethys and Atlantic Oceans^1–5^ (Fig. 1). In particular, four prominent black shale horizons (113/Jacob, Kilian, Urbino/Paquier, and Leenhardt equivalent levels) have been identified as the sedimentary expression of OAE1b^4^. OAE1b is characterized by (1) an exceptionally long duration (~ 3.8 Myr) with intermittent occurrences of oxygen-depleted bottom water conditions, (2) a global carbon-cycle perturbation, and (3) a major marine biotic turnover^4^. In particular, planktonic foraminifera experienced one of the most significant turnovers in their evolutionary history, where large, heavily calcified planktonic foraminifera of the Aptian were replaced by small, weakly calcified taxa characteristic of the Albian^4,6–8^.

Since ^40^Ar–^39^Ar ages of the Southern Kerguelen Plateau basalt (Fig. 1) (109.2–119.0 Ma)^9^ roughly correspond to the duration of OAE1b (~ 110.5–114.5 Ma), volcanic episodes associated with the break-up of Gondwana have been suggested as the trigger of OAE1b^1,3,5^. However, the issue remains debated because of the large chronological uncertainties of the ^40^Ar–^39^Ar ages of the basaltic rocks and the ages of the sedimentary sequences. Marine osmium (Os) isotopic records (^187^Os/^188^Os) reflect the balance between the continental Os flux (^187^Os/^188^Os ≈ 1.0–1.5) and mantle/hydrothermal and extraterrestrial Os fluxes (^187^Os/^188^Os ≈ 0.12–0.13) to the global ocean^10^. Thus, ^187^Os/^188^Os values of palaeo-seawater preserved in sedimentary rocks represent a robust proxy to constrain the timing of massive input of unradiogenic Os through submarine hydrothermal or volcanic eruption. Since the estimated residence time of Os in the modern ocean is 10^4^–10^5^ years, that is longer than the timescale of modern oceanic circulation^10,11^, seawater ^187^Os/^188^Os values are relatively constant throughout the global oceans. Here, we present Late Aptian to Early Albian palaeo-marine Os isotopic variations in the Poggio le Guaine (PLG) record (central Italy, deposited in the central to western Tethys) (Fig. 1 and Supplementary Fig. S1) and Deep Sea Drilling Program (DSDP) Site 463 (western Mid-Pacific mountains, central Pacific Ocean) (Fig. 1 and Supplementary Fig. S2) to constrain the timing of extensive volcanic episodes during OAE1b. For the Os isotopic analysis of organic-rich sedimentary rock samples of PLG section, we preferentially used borehole core^12^ samples drilled near the PLG section because initial Os isotopic information of these rocks are easily altered by weathering.

### Lithological description

#### PLG records.

**Figure 2:**
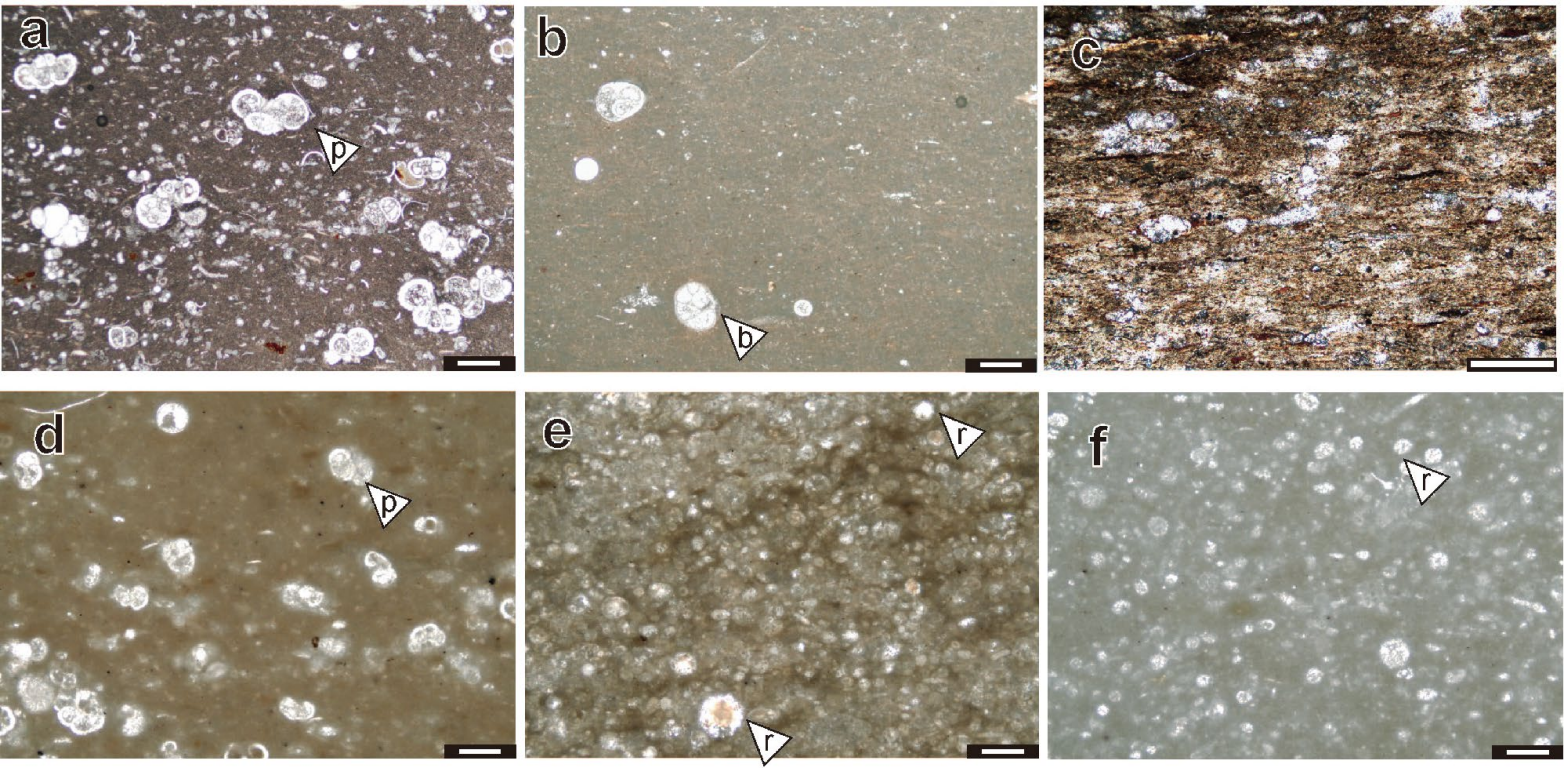
Thin sectional images of PLG section and DSDP Site 463. (**a**) limestone at 12 m, PLG section, (**b**) marlstone at 16 m, PLG section, (**c**) black shale (Urbino level) at 25.28 m, PLG section, (**d**) DSDP Site 463, core 62, Sect. 1, 80–82 cm (538.3 mbsf), (**e**) DSDP Site 463, core 62, Sect. 1, 133–138 cm (529.3 mbsf : Kilian equivalent interval), (**f**) DSDP Site 463, core 59, Sect. 2, 116–118 cm (521.16 mbsf). *p* planktonic foraminifera, *b* benthic foraminifera, *r* radiolaria. All scale bars are 200 μm.

**Figure 3:**
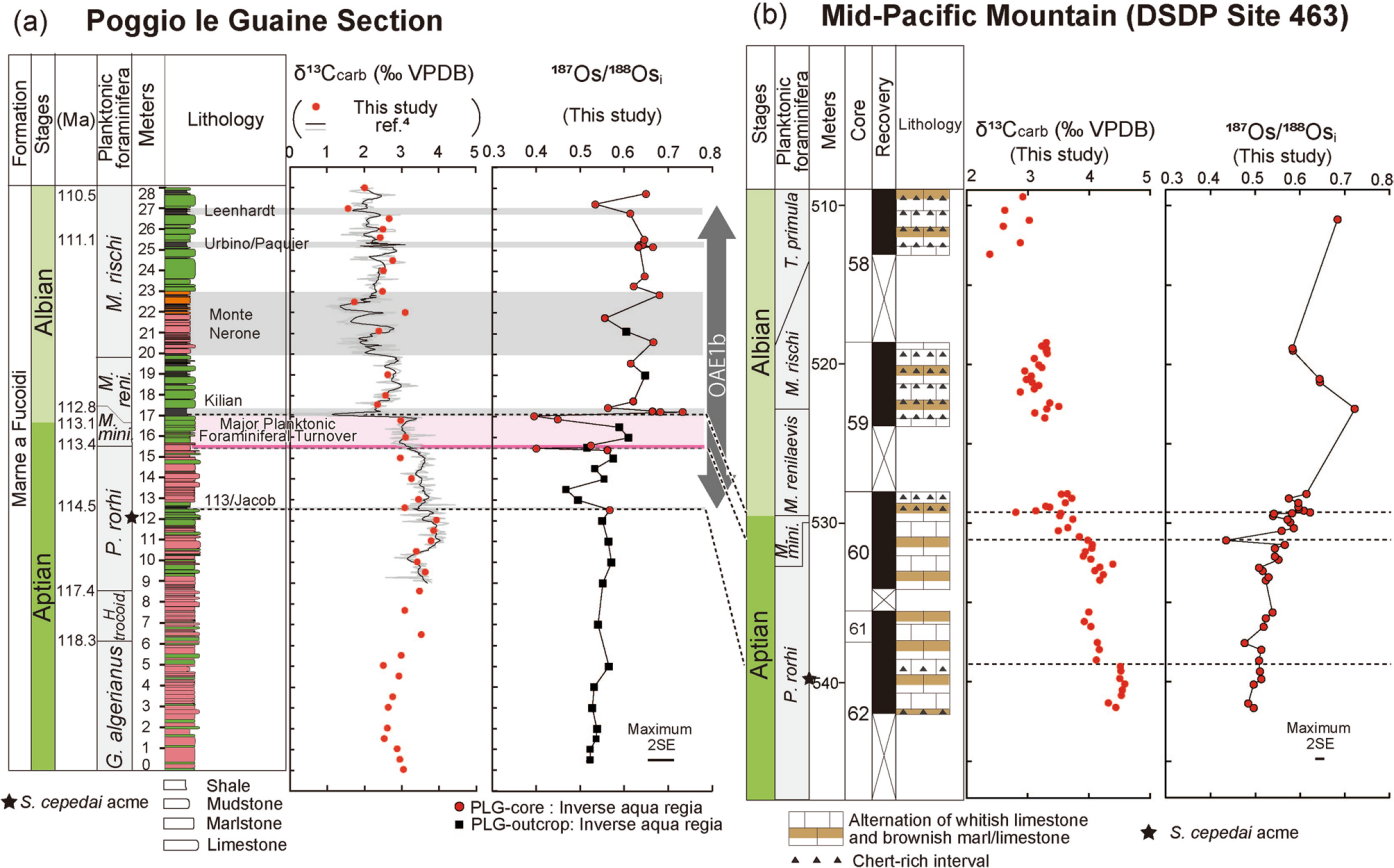
Carbon and Os isotopic variations at (**a**) the PLG section and (**b**) DSDP Site 463. Grey and black lines in the δ^13^C_carb_ profile in (**a**) are the δ^13^C_carb_ values of ref^4^ Dashed horizontal lines mark the 113/Jacob equivalent level, onset of the planktonic foraminiferal turnover, and the Kilian equivalent level. The pink-shaded band in (**a**) highlights the planktonic foraminiferal turnover. Grey shaded areas in (**a**) indicate prominent shale horizons. Star marks the *S. cepedai* acme. The lithology of PLG section consists of shale, mudstone, marlstone, and limestone rich in planktonic foraminifera and calcareous nannofossils. Color of lithological column in (**a**) represents the color of sediments. The lithology of DSDP Site 463 consists of alternation of white/brownish limestone and brownish marlstone with minor chert layer. Triangles in (**b**) represents chert-rich interval. *G.* *Globigerinelloides*, *H.* *Hedbergella*, *P.* *Paraticinella*, *M.* *Microhedbergella, S.* *Schackoina*.

**Figure 4:**
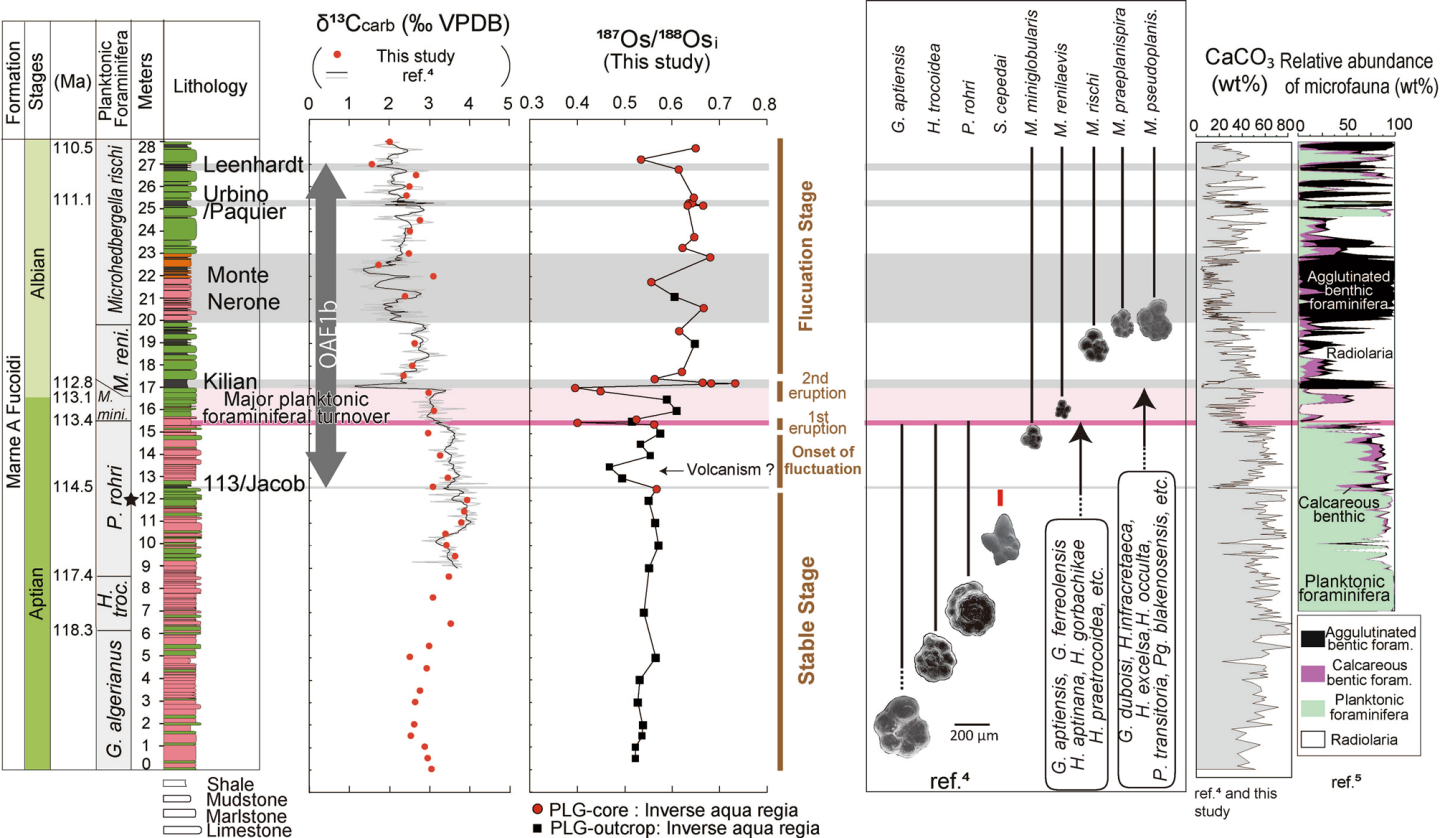
Sable carbon isotopic record, Os isotopic record, major changes in planktonic foraminiferal assemblages^4^, changes in CaCO_3_ content (from this study, refs^4,5^), and relative microfauna abundance^4,5^ at the PLG record.

The PLG section (Fig. 1, Supplementary Figs. S1, S3 and Supplementary Note 1.1) is one of the most continuous sedimentary successions encompassing OAE1b^4^. Since coarse silicate fragments are rare throughout this section (Fig. 2a–c), this sedimentary sequence was considered deposited in a pelagic environment. The pre-OAE1b interval mainly comprises reddish or olive-grey argillaceous limestone and marlstone enriched in heavily calcified large planktonic foraminifera (Figs. 2a, 3a, 4 and Supplementary Fig. S3a), whereas the OAE1b interval is characterized by a cyclic alternation of olive to greenish-grey marlstone/mudstone and black shale (Figs. 3a, 4 and Supplementary Fig. S3b,c) where the size and abundance of planktonic foraminifera decreased (Figs. 2b, 4). The 113/Jacob, Kilian, Urbino/Paquier, and Leenhardt equivalent levels are the regional sedimentary expression of OAE1b sub-events^4^ (Figs. 2c, 3 and Supplementary Figs. S3, S4). The 113/Jacob equivalent level is the first organic-rich sediment during OAE1b. A distinctive sequence of alternating reddish and black shales, called Monte Nerone interval, occurs between the Kilian and Urbino/Paquier equivalent levels (Fig. 3). Above 15.42 m stratigraphic level in the section (pink line in Figs. 3, 4), small, weakly calcified Albian planktonic foraminiferal taxa appear followed by the abrupt decrease in the number of large (> 250 μm), heavily calcified Aptian planktonic foraminifera^4^. The heavily calcified planktonic foraminifera characteristic of Aptian never appeared above the thick black shale horizon called Kilian level (16.97–17.37 m)^4^ (Fig. 4 and Supplementary Fig. S3b). This major planktonic foraminiferal species turnover is accompanied by a significant increase in the radiolarian abundance^4^ and a decrease in calcium carbonate (CaCO_3_) content (Fig. 4). The Kilian equivalent level is punctuated by a major negative carbon isotopic excursion (CIE) (Fig. 3) coupled with a marked decrease of CaCO_3_ content and the dominance of agglutinated foraminiferal taxa^4^ (Fig. 4).

The drilling site of PLG-core is located 400 m northwest of the PLG Section^12^. The Upper Aptian–Lower Albian sedimentary and biostratigraphic record of PLG core is well correlated to that of PLG section. Therefore, the geochemical data of outcrop and borehole core can be confidently combined.

#### DSDP Site 463

The Lower Cretaceous record at DSDP Site 463 (Fig. 1 and Supplementary Fig. S2) mainly comprises varicoloured limestone and marlstone with minor chert layer (Fig. 2d–f, Supplementary Fig. S5, and Supplementary Note 1.2). The Upper Aptian to Lower Albian sedimentary sequence lacks organic-rich sediments^13^ and does not exhibit any marked lithological changes.

## Results

We measured carbonate carbon isotopic compositions (δ^13^C_carb_) of limestone and marlstone samples of PLG section and DSDP Site 463 for stratigraphic correlation (see Method 4) (Fig. 3, Supplementary Fig. S6 and Tables S1, S2, and Supplementary Note 2). The δ^13^C_carb_ values of PLG section were 1.7–3.4‰ (Fig. 3a) and consistent with those of presented in ref^4^. Although the δ^13^C_carb_ values of DSDP Site 463 (2.4–4.6‰; Fig. 3b) are higher than those ones at PLG section by ~ 1‰, their temporal variation is quite similar. The δ^13^C_carb_ record at DSDP Site 463 shows a negative CIE at ~ 539 m below seafloor (mbsf), just above the *Schackoina cepedai* acme at 539.8 mbsf (Fig. 3b and Supplementary Note 3). Since the *S. cepedai* acme at the PLG section falls ~ 1.2 m below the 113/Jacob equivalent level (the first organic-rich horizon in the Tethys region during OAE1b) which is marked by a negative CIE^4^ (Fig. 3), we correlate the CIE in the Pacific Ocean with the 113/Jacob equivalent level (Fig. 3). A more distinct negative CIE (~ 1‰) occurs at 529.33 mbsf, just above the lowest occurrence of *Microhedbergella renilaevis* (529.56 mbsf). Since these features are consistent with those recorded across the Kilian equivalent level, which records the demise of the Aptian planktonic foraminifera in the PLG section, we correlate this CIE at DSDP Site 463 to that of the Kilian level (Fig. 3).

At the PLG section, the total organic carbon content (TOC) values of pre-OAE1b ranges from 0.01 to 0.04% and averages 0.03% (see Methods 5) (Supplementary Fig. S7 and Table S3). The TOC values of black shale horizons during OAE1b are higher than other horizons. In particular, TOC values across the Jacob and Urbino equivalent levels show extremely high values (up to 8%). Other black shale intervals (i.e., Kilian equivalent level, Monte Nerone interval, and Leenhardt equivalent level) reveal lower concentrations of TOC (up to 0.65%). TOC values of sedimentary rocks from the DSDP Site 463 are 0.04–0.12% and we could not find organic rich intervals (Supplementary Table S4).

We conducted Re-Os analysis of limestone, marlstone, mudstone, and black shale samples collected from the PLG record (outcrop and core) and limestone and marlstone samples of the DSDP Site 463 (see Method 6) (Fig. 3, Supplementary Figs. S8, S9 and Tables S5, S6 and Supplementary Note 4). Considering the positive correlations between TOC and Re, Os concentrations throughout the PLG section, these elements are derived from the hydrogenous fraction associated with organic matters (Supplementary Fig. S10)^14,15^. Since there are no clear relationships between other elements (e.g. Fe and Mn), the contribution of Os and Re derived from other fractions (such as ferromanganese oxides)^16^ is considered minor (Supplementary Fig. S11 and Table S7). Most of the PLG core samples were treated with aqua regia digestion. However, for some samples of PLG section, we applied both CrO_3_-H_2_SO_4_ and inverse aqua regia to check whether our method extract hydrogenous fraction or not. On the basis of these results, we could not find any significant differences in ^187^Os/^188^Os_i_ (see Method 6) (Supplementary Figs. S12, S13 and Supplementary Note 4), which supports our approach to successfully extract the hydrogenous information.

### Osmium isotopic records across the Aptian–Albian boundary

**Figure 5:**
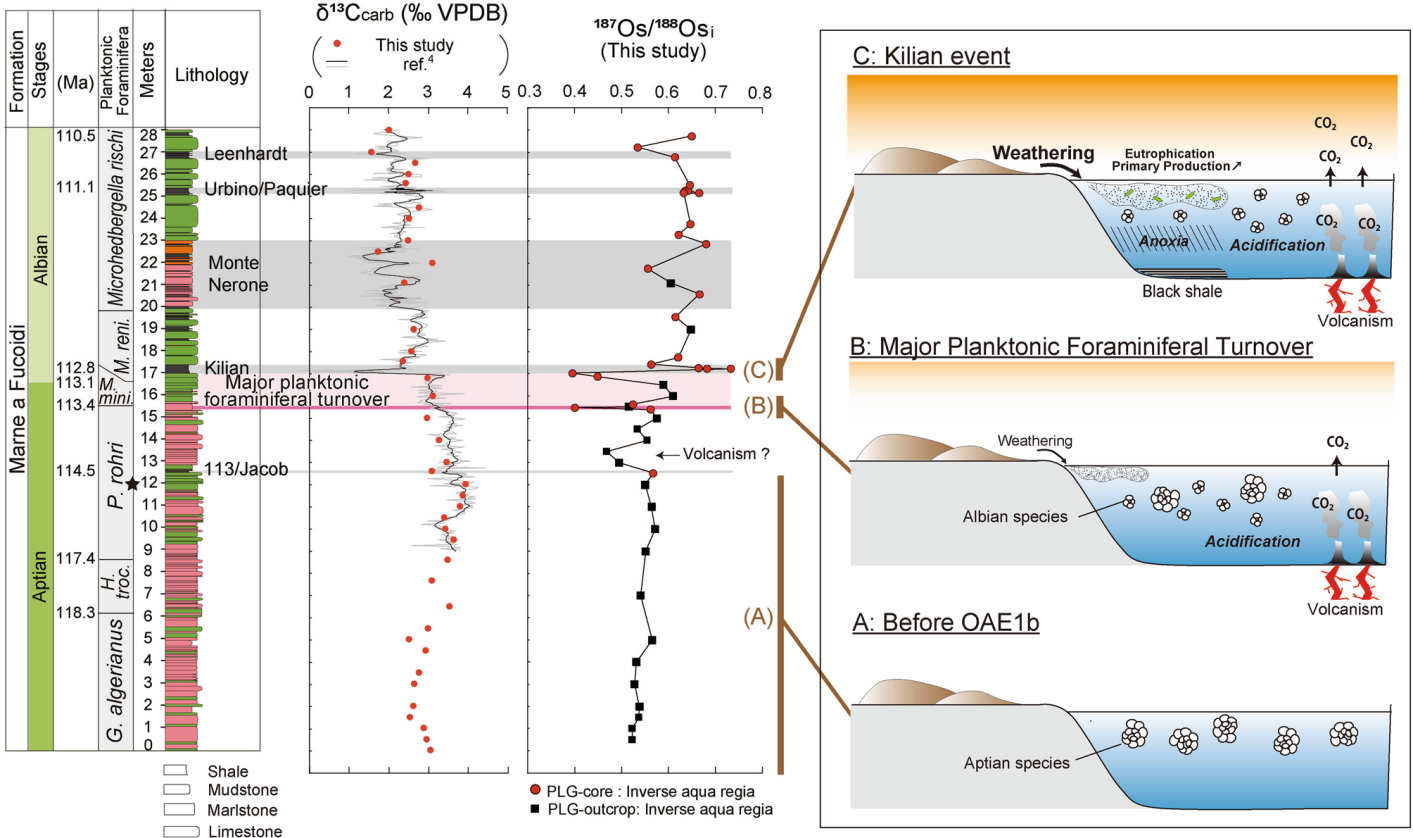
Interpretation of Os isotopic variations and environmental changes during OAE1b in the context of the lithological, geochemical, and biological profiles reported in Fig. 4. (**a**) Oceanic conditions during the pre-OAE1b interval; (**b**) the first volcanic eruption at the extinction level; and (**c**) the second volcanic eruption at the Kilian level.

In the pre-OAE1b interval, ^187^Os/^188^Os_i_ values (i.e., initial ^187^Os/^188^Os values, corrected for the radioactive decay of ^187^Re to ^187^Os, see methods) range from 0.52 to 0.57 (average 0.54) in the PLG section and from 0.49 to 0.51 (average 0.50) at DSDP Site 463 (Figs. 3, 4, 5). These values are slightly lower than the pre-OAE1a values of the Lower Aptian (~ 0.7)^17^ and pre-OAE2 values of the Upper Cenomanian (~ 0.7)^18^. Since several seawater temperature proxies (belemnite oxygen isotopic ratios, the organic palaeothermometer TEX_86_, and the existence of glendonites at higher latitudes) suggest a cooler climate during the Late Aptian than during the Early Aptian and Late Cenomanian^19–21^, these low ^187^Os/^188^Os_i_ values may reflect weak continental weathering under cool climatic conditions.

The ^187^Os/^188^Os_i_ values began to fluctuate immediately after the onset of OAE1b (i.e. the deposition of the Jacob level) in the PLG section. The first sharp negative spike of ^187^Os/^188^Os_i_ values appears few-cm below the major planktonic foraminiferal turnover (15.48 m; pink line in Fig. 3) at the PLG section. Similar Os isotopic spike can be also recognized at the DSDP Site 463 (531.08 mbsf: Fig. 3a). The ^187^Os/^188^Os_i_ values again sharply decline in the lower part of the Kilian level in the PLG section, then rapidly increase to 0.73 (Fig. 3a). Although the pattern of ^187^Os/^188^Os_i_ variations is quite similar between the two sites, the amplitude of ^187^Os/^188^Os_i_ variations at the Kilian equivalent level at DSDP Site 463 (0.54–0.62) is much smaller than that in the PLG Section (0.40–0.73) (Fig. 3b). Considering the long residence time of Os in the present ocean (10–100 kyr)^10,11^, the significant difference in the range of ^187^Os/^188^Os_i_ values between these two localities is a conundrum. Borehole core samples from both sites are fresh, and the influence of weathering seems insignificant. We consider active bioturbation or a small hiatus around the Kilian equivalent horizon at DSDP Site 463 to be the most probable explanation of the decreased ^187^Os/^188^Os_i_ variations there. Indeed, the amplitude of the negative δ^13^C_carb_ spike at the Kilian equivalent level of DSDP Site 463 (~ 1‰) is smaller than the one at PLG section (> 2‰), which also implies the mixing of sediments or existence of minor hiatus during Kilian event. The sedimentary expression of the Kilian level at the PLG section is a 40 cm-thick black shale with minor bioturbation^4^ whereas that of DSDP Site 463 is bioturbated lime/marlstone thinner than 20 cm (Fig. 2e and Supplementary Fig. S5). Therefore, we consider the Os isotopic variations in the PLG section to be less disturbed and to better reflect the initial Os isotopic signature of seawater than those at the DSDP Site 463.

The two sharp declines of ^187^Os/^188^Os_i_ values to ~ 0.4 (Figs. 3 and 4) can be explained either by (1) rapid declines in continental weathering, (2) large meteorite impacts, or (3) increases in hydrothermal activity. Here, we apply a simple box model calculation to evaluate these possibilities (see Method 7). The first one requires an abrupt 35% decrease in radiogenic continental Os within a few hundred thousand years (Supplementary Table S8), probably through rapid cooling events. However, no such abrupt cooling of the climate has yet been reported. Instead, intensive warming was reported at the Kilian equivalent level in the Atlantic Ocean^19,21^, which, on the contrary, would have accelerated chemical weathering. Decreased continental weathering therefore seems unlikely as the cause of the decreased ^187^Os/^188^Os_i_ values. Although a meteorite impact could explain the sharp decline of ^187^Os/^188^Os_i_ values, neither a large meteorite crater nor tektites have been reported in correspondence of this stratigraphic interval. Therefore, extensive volcanic events on the Southern Kerguelen Plateau^9^, which could have released a large amount of unradiogenic Os through hydrothermal activities, seems the most reasonable explanation. Our box-model calculation shows Kerguelen volcanic episodes at that time promoted a 60–90% increase in the unradiogenic Os flux through hydrothermal activities. The ^187^Os/^188^Os_i_ values abruptly increase from 0.40 to 0.73 in the upper part of the Kilian level in the PLG section (Fig. 3). Based on our box-model calculation, this abrupt increase in ^187^Os/^188^Os_i_ values can be explained by either a 41% decrease in the hydrothermal Os flux associated with the oceanic crustal production or a 67% increase in the continental weathering rate. Considering the intensive warming during the Kilian event^19,22^, a rapid increase in the radiogenic Os flux through continental weathering appears plausible. Based on the characteristics of Kerguelen lava flows, a large part of the volcanic plateau was emplaced subaerially, and the submarine eruption was short lived^23^. We interpret this positive ^187^Os/^188^Os_i_ anomaly as a later eruptive phase when active CO_2_ degassing via subaerial eruptions enhanced continental weathering and weakened the hydrothermal input of unradiogenic Os. Similar positive ^187^Os/^188^Os_i_ anomalies have not been reported yet for other Cretaceous OAEs (OAE1a^17^ and OAE2^18^). This might be ascribed to the entirely submarine volcanic eruptions of the Ontong Java Plateau and Caribbean Plateau, which triggered OAE1a and OAE2, respectively, and continuously supplied a large amount of unradiogenic Os through hydrothermal activities throughout the eruptions.

The ^187^Os/^188^Os_i_ values above the Kilian equivalent level (average 0.63 both in the PLG section and at the DSDP Site 463) are higher than the pre-OAE1b background values (0.54 in the PLG section and 0.50 at the DSDP Site 463) (Fig. 3). Planktonic foraminifera oxygen isotopic records in the Atlantic Ocean (Ocean Drilling Program Site 1,049) indicate a rise in seawater temperature after the Aptian–Albian transition relative to the pre-OAE1b interval^24^. Furthermore, the disappearance of glendonite from Arctic sediments^21^ supports a warming climate after the Aptian–Albian boundary, which could have sustained high continental weathering rates and high marine ^187^Os/^188^Os_i_ values. The radiogenic strontium isotopic ratio (^87^Sr/^86^Sr) of seawater also continuously declined from the Early to Late Aptian, with an incipient increase above the Aptian–Albian boundary^25^. Such trends suggest a larger contribution of continental weathering during OAE1b (Early Albian) than during the pre-OAE1b interval (Late Aptian). After the initial sharp declines of ^187^Os/^188^Os_i_ values to ~ 0.4, ^187^Os/^188^Os_i_ values fluctuated throughout OAE1b from 0.53 to 0.68 in the PLG section and from 0.58 to 0.71 at DSDP Site 463 (Fig. 3). Considering the protracted volcanic eruptions of the Southern Kerguelen Plateau^9^, extended minor eruptions after the main volcanic pulse might have caused fluctuations of the continental weathering rate and/or the hydrothermal Os influx, eventually contributing to the prolonged perturbation of the Os cycle (Fig. 3).

### Major planktonic foraminiferal turnover

The two volcanic Os isotopic signals correspond to the major planktonic foraminiferal turnover interval characterized by the extinction of large, heavily ornamented calcified planktonic foraminifera and the speciation of smaller thin, weakly calcified species that lack ornamentation in the PLG Section^4,8^. These volcanic horizons are accompanied by abrupt decreases in CaCO_3_ content and the poor preservation of calcareous microfossil tests, which means the dissolution of CaCO_3_ on the seafloor. Moreover, the relative abundance of agglutinated benthic foraminifera, which is resilient to ocean acidification, increased at the same time in the Tethys region^4^ (Fig. 4). These features suggest a shallowing of the carbonate compensation depth (CCD)^26–28^ at these volcanic horizons. A massive input of volcanic CO_2_ could have prompted ocean acidification, potentially leading to dwarfism of planktonic foraminifera and shallowing of the CCD. Positive relationships among pH, test thickness, and growth rates of foraminifera have been documented in laboratory experiments and geological observations^29–31^. Therefore, the drastic turnover and reduction in the size of planktonic foraminifera may reflect a decline of pH caused by the massive release of volcanic CO_2_ (Fig. 4). Indeed, since acidified condition prevents the growth of ornamentation and spines of foraminifera^28,31,32^, the lack of ornamentation in smaller Albian species^4,8^ might reflect adaptation to the acidified oceanic condition. No major extinction of calcareous nannoplankton has been reported during OAE1b. Marine organisms with extremely small calcified tests might have been less influenced by ocean acidification^33,34^, probably, because of their more efficient proton pumping systems in the carbon pools inside their cells relative to larger calcareous shell-forming organisms^35^.

The large negative δ^13^C_carb_ spike at the Kilian equivalent level implies a more intensive volcanic input of ^13^C-depleted CO_2_ than during the first volcanic pulse at the beginning of the biotic turnover (Fig. 5). The resulting intensive climatic warming and subsequent massive input of nutrients into the ocean through continental weathering could have triggered increased primary production, leading to oceanic anoxia in the Tethyan and Atlantic Oceans (Fig. 5).

No evidence of an O_2_-depleted environment has yet been reported for the first volcanic pulse. Furthermore, O_2_-depleted conditions during the Kilian event (i.e., during the latest stage of the biotic turnover) were limited to the Tethyan and the Atlantic Oceans. These facts might imply that oceanic anoxia was not the direct trigger of the major marine biota turnover during OAE1b and possibly, the ocean acidification induced by volcanic events could have contributed to the biotic crisis. This conclusion suggests that oceanic anoxia and the extinction of marine biota are different phenomena that should be separately discussed.

## Summary

Here, we presented continuous marine Os isotopic variation across the Aptian–Albian boundary at Tethyan pelagic section (PLG section) and Pacific sediments (DSDP Site 463). We found two sharp negative shifts around the Aptian–Albian boundary, which suggest submarine volcanic eruptions. Since these intervals corresponds to the major planktonic foraminiferal species turnover and neither of them correspond to global oceanic anoxia, we concluded this turnover was likely caused by ocean acidification triggered by two major volcanic events.

## Methods

### Micropalaeontological studies

All samples were analysed for planktonic foraminifera. On average, 8 g of rock were processed for each sample. Owing to the hard lithology, all samples were mechanically disaggregated into small fragments (3–8 mm) and treated following the cold acetolysis technique of ref^36^ by sieving through a 63 μm mesh and drying at 50 °C. The cold acetolysis method enables the extraction of easily identifiable foraminifera. This technique offers accurate taxonomic determination and detailed analysis of planktonic foraminiferal assemblages, allowing a more precise placement of several bioevents and zonal boundaries.

Moderately to well-preserved planktonic foraminifera were present in almost all samples. Planktonic foraminifera from the washed residues of each size fraction (63–100, 100–125, 125–150, 150–180, 180–250, 250–355, 355–500, and > 500 μm were separately studied under a stereomicroscope. Taxonomic concepts for genera and species identification in this study mainly follow ref^6^ and the online Mikrotax database (https://mikrotax.org/pforams/index.php?dir=pf_mesozoic), where the type materials for most species are illustrated by recent scanning electron microscopy images^37^. The biostratigraphic framework used in this study is after ref^6,38^, and references therein.

### Measurement of CaCO_3_ content

The sedimentary rock samples of PLG section were crushed and ground into fine powder in an agate mortar. Powdered samples were dissolved in 10% vol. HCl and CaCO_3_ content were obtained by measuring CO_2_ volume using a Dietrich–Frühling calcimeter. Detailed procedure is the same of ref^4^.^.^

### Sample trimming

The surfaces of the collected sediment samples were trimmed with a spatula and weathered parts were removed. The samples were then crushed into coarse fragments, from which fresh fragments were collected. These samples were washed in deionized water in an ultrasonic bath and dried in an oven at 60 °C for at least 12 h. The dried samples were crushed and ground to a fine powder using an agate mill. These powdered samples were used for stable carbon isotopic analyses, total organic carbon measurements to determine the host phases of Os and Re, and finally for Re-Os isotopic analyses.

### Stable carbon and oxygen isotopic compositions of carbonate

Carbonate stable carbon (δ^13^C_carb_) and oxygen isotopic compositions (δ^18^O_carb_) were measured in 39 samples from the PLG section and 68 samples from DSDP Site 463 using a GV IsoPrime instrument at the Japan Agency for Marine-Earth Science and Technology (JAMSTEC). The isotopic compositions are expressed in delta notation as per-mil variations relative to Vienna Peedee Belemnite (VPDB). Analytical errors (1σ) on δ^13^C_carb_ and δ^18^O_carb_ were estimated to be within 0.1‰ and 0.2‰, respectively on the basis of repeated measurements of in-house standard materials. The isotopic analytical method is detailed by ref^39^

### Stable carbon isotopic compositions of organic matter and total organic carbon contents (TOC)

We determined total organic carbon contents (TOC) and organic stable carbon isotopic compositions (δ^13^C_org_) for sedimentary rock samples from the PLG section and 11 sedimentary rock samples from the PLG core. Powdered and weighed samples were decalcified with 2 M HCl, rinsed with purified water using a MilliQ Water purification system. After evaporation to dryness, these samples were weighted to calculate the change in weight during decalcification. These samples were wrapped in a Sn cup for measurements. Carbon contents and δ^13^C_org_ were measured at JAMSTEC via on-line system of isotope-ratio mass spectrometry (DeltaPlus XP; Finnigan, Waltham, MA, USA) coupled to a Flash EA 1,112 Automatic Elemental Analyzer through a ConFlo III interface^40^ Analytical errors (1σ) on δ^13^C_org_ is better than 0.37‰ based on repeated measurements of the in-house standard (L-Tyrosine).

### Rhenium and osmium analysis

We used CrO_3_-H_2_SO_4_ digestion^41^ and inverse aqua regia digestion methods to extract Re and Os from the samples. Samples for Re-Os analyses varied from 0.1 to 1 g depending on the expected concentration of Os in the samples. In fact, organic-rich black shale samples generally show high Os and Re concentration and marl/limestone samples with low TOC shows lower Os and Re concentrations. After spiking using ^190^Os- and ^185^Re-rich solutions, each sample was sealed in a Carius tube^42^ with 4 mL of CrO_3_-H_2_SO_4_ (0.2 g CrO_3_ per 1 mL of 4 N H_2_SO_4_) or inverse aqua regia (mixture of 1 mL of 30 wt% HCl and 3 mL of 68 wt% HNO_3_) digestion solutions. The sample solutions were heated at 240 °C for 48 h. Through this process, Os and Re were completely extracted from the samples as Os(VIII)O_4_ and Re(VII)O_4_^–^, respectively, and isotopic equilibrium between the spike and sample was achieved. The supernatant (leachate) was separated from the residue by centrifugation. After removing the residue, Os was separated from the leachate with 3 mL of carbon tetrachloride (CCl_4_) in three successive extractions. The volatile Os(VIII)O_4_ was reduced to non-volatile Os(IV)Br_6_^2–^ by adding 3 mL of 9 N HBr. Extracted Os was purified by micro-distillation^43^. Re was separated from the leachate thorough following two steps: (1) 2 mL of Bio-Rad AG1-X8 anion exchange resin (100–200 mesh) and (2) 0.3 mL of Bio-Rad AG1-X8 anion exchange resin (100–200 mesh).

Os abundances and isotopic compositions were determined by negative thermal ionization mass spectrometry (Thermal Electron TRITON) (ref^44^ and references therein) at JAMSTEC (Japan) and Re abundances and isotopic compositions by quadrupole inductively coupled plasma mass spectrometry (iCapQ) at JAMSTEC using in-house Re standards. All data were corrected for procedural blanks, whose respective averages were 0.57 pg Os and 5.4 pg Re. The average of ^187^Os/^188^Os of procedural blank is 0.13. Instrumental reproducibility (standard error) was monitored based on replicate analyses of the in-house standard for ^187^Os/^188^Os = 0.106838 ± 0.000015 (2σ)^45^.

The ^187^Re decays to ^187^Os in sediments with a decay constant of 1.666 × 10^–11^ yr^–l^^46^. Therefore, the initial ^187^Os/^188^Os of sediments (^187^Os/^188^Os_i_) was calculated as:

1 $${}^{187}Os/{}{}^{188}{Os}_{i}={}{}^{187}Os/{}{}^{188}{Os}_{m}-[exp\left\{\lambda \times age\left(yr\right)\right\}-1] \times {}{}^{187}Re/{}{}^{188}{Os}_{m}$$

Here, *λ* is the decay constant of 1.666 × 10^–11^ year^–l^ and the subscript ‘m’ indicates measured values. For the sedimentary rock samples from the PLG section and PLG core, ages used for the correction were 119 Ma from 0 to 6 m in the section, 117 Ma from 6 to 9 m, 115 Ma from 9 to 12 m, 114 Ma from 12 to 15 m, 113 Ma from 15 to 20 m, 112 Ma from 20 to 23 m, and 111 Ma from 23 to 26 m. In the DSDP Site 463 core, ages used for the correction were 114 Ma for DSDP Site 463, cores 62–61 and 113 Ma for DSDP Site 463, cores 61–58 (Supplementary Tables S5, S6). These ages were determined based on refs^4,47^.

### Calculation of Os fluxes using a simple box model

We calculated the changes in the Os flux through hydrothermal activities and continental weathering using a zero-dimensional box model based on ref^17^. This model assumes the ocean to be a unique Os reservoir, and its Os content and isotopic composition to reflect the balance between continental input, hydrothermal input related to oceanic crustal production and volcanic eruptions at the Southern Kerguelen Plateau, extraterrestrial input, and a sedimentary sink. These relationships are described as:

2 $$\frac{d{M}_{ocean}}{dt}={F}_{cont}+{F}_{hydr}+{F}_{cosm}+{F}_{Kerg}-{F}_{sed}$$

3 $$\frac{d({M}_{ocean}{R}_{ocean})}{dt}={F}_{cont}{R}_{cont}+{F}_{hydr}{R}_{hydr}+{F}_{cosm}{R}_{cosm}+{F}_{Kerg}{R}_{Kerg}-{F}_{sed}{R}_{sed}$$

where *M*, *F*, and *R* indicate the amount, flux, and isotopic ratio (^187^Os/^188^Os) of Os, and the subscripts ‘ocean’, ‘cont’, ‘hydr’, ‘cosm’, ‘Kerg’, and ‘sed’ represent the oceanic reservoir, continental input, hydrothermal input, extraterrestrial input, input from the Southern Kerguelen Plateau, and sedimentary output, respectively. Since the isotopic fractionation of Os is negligible between sediments and seawater, we assumed that *R*_sed_ coincides with *R*_ocean_. Then, the above equations can be combined as:

4 $$\frac{d{R}_{ocean}}{dt}=\frac{[{F}_{cont}\left({R}_{cont}-{R}_{ocean}\right)+{F}_{hydr}\left({R}_{hydr}-{R}_{ocean}\right)+{F}_{cosm}({R}_{cosm}-{R}_{ocean})]}{{M}_{ocean}}$$

Here, we used the present-day values of *F*_cont_ = 295 t/kyr, *R*_cont_ = 1.54^10,48^, *R*_hydr_ = 0.126^49^ *F*_cosm_ = 17.6 t/kyr, and *R*_cosm_ = 0.126^10,49^ for the steady background conditions below the Jacob level. The steady background oceanic condition is *R*_ocean_ = 0.54, and we set *F*_hydr_ to 532.7 t/kyr to match this value because several studies have suggested that hydrothermal activities associated with the production of oceanic crust in the Cretaceous were more active than today (e.g., ref^50^). For the output parameters, we assumed that *R*_sed_ varies proportionally to *R*_ocean_, and set the coefficient of proportionality at 0.056 following ref^17^. Since there are no Os isotopic data available for Southern Kerguelen Plateau basalts, we used the isotopic composition of Kerguelen Archipelago lavas (^187^Os/^188^Os = 0.16)^51,52^ for *R*_Kerg_. At steady state, *F*_Kerg_ is considered to be 0 t/kyr. Only Os isotopic values from the PLG section were used for *R*_ocean_ because the PLG section is more continuous and spans a longer time interval than DSDP Site 463. Detailed parameter values throughout the cores are listed in Supplementary Table S8.

### Major elemental composition of bulk sedimentary rock samples

The powdered samples and lithium tetraborate flux (Li_2_B_4_O_7_, MERCK) were heated at 110 °C for more than 12 h. Dried powdered samples were weighed and heated at 950 °C for 7 h. After measurement of their weight to calculate loss of ignition, about 0.4 g samples and exactly ten-times larger amount of Li_2_B_4_O_7_ were mixed and heated to make glass beads. They were analyzed by ZSX Primus II XRF spectrometer (Rigaku) installed in Atmosphere and Ocean Research Institute (AORI), the University of Tokyo, Japan to determine major elemental composition.

## Supplementary information

Supplementary file1 (PDF 2366 kb)

## References

[1] Leckie RM, Bralower TJ, Cashman R (2002). Oceanic anoxic events and plankton evolution: Biotic response to tectonic forcing during the mid-Cretaceous. Paleoceanography.

[2] Herrle JO (2004). High-resolution carbon isotope records of the Aptian to Lower Albian from SE France and the Mazagan Plateau (DSDP Site 545): a stratigraphic tool for paleoceanographic and paleobiologic reconstruction. Earth Planet. Sci. Lett..

[3] Trabucho-Alexandre J (2011). The sedimentary expression of oceanic anoxic event 1b in the North Atlantic. Sedimentology.

[4] Coccioni R (2014). The neglected history of Oceanic Anoxic Event 1b: insights and new data from the Poggio le Guaine section (Umbria–Marche Basin). Stratigraphy.

[5] Sabatino N (2018). Mercury anomalies in upper Aptian-lower Albian sediments from the Tethys realm. Palaeogeogr. Palaeoclimatol. Palaeoecol..

[6] Huber BT, Leckie RM (2011). Planktic foraminiferal species turnover across deep-sea Aptian/Albian boundary sections. J. Foraminiferal Res..

[7] Petrizzo MR, Huber BT, Gale AS, Barchetta A, Jenkyns HC (2012). Abrupt planktic foraminiferal turnover across the Niveau Kilian at Col de Pré-Guittard (Vocontian Basin, southeast France): new criteria for defining the Aptian/Albian boundary. Newsl. Stratigr..

[8] Ferraro S (2020). Morphometric response of late Aptian planktonic foraminiferal communities to environmental changes: A case study of Paraticinella rohri at Poggio le Guaine (central Italy). Palaeogeogr. Palaeoclimatol. Palaeoecol..

[9] Coffin MF (2002). Kerguelen hotspot magma output since 130 Ma. J. Petrol..

[10] Levasseur S, Birck JL, Allègre CJ (1999). The osmium riverine flux and the oceanic mass balance of osmium. Earth Planet. Sci. Lett..

[11] Levasseur S, Birck JL, Allègre CJ (1998). Direct measurement of femtomoles of osmium and the ^187^Os/^186^Os ratio in seawater. Science.

[12] Coccioni R (2012). Umbria-Marche Basin, Central Italy: A reference section for the Aptian-Albian interval at low latitudes. Sci. Drilling.

[13] Price GD (2003). New constraints upon isotope variation during the early Cretaceous (Barremian–Cenomanian) from the Pacific Ocean. Geol. Mag..

[14] Ravizza G, Turekian KK, Hay BJ (1991). The geochemistry of rhenium and osmium in recent sediments from the Black Sea. Geochim. Cosmochim. Acta.

[15] Creaser RA, Sannigrahi P, Chacko T, Selby D (2002). Further evaluation of the Re-Os geochronometer in organic-rich sedimentary rocks: A test of hydrocarbon maturation effects in the Exshaw Formation, Western Canada Sedimentary Basin. Geochim. Cosmochim. Acta.

[16] Yamashita Y, Takahashi Y, Haba H, Enomoto S, Shimizu H (2007). Comparison of reductive accumulation of Re and Os in seawater–sediment systems. Geochim. Cosmochim. Acta.

[17] Tejada MLG (2009). Ontong Java Plateau eruption as a trigger for the early Aptian oceanic anoxic event. Geology.

[18] Turgeon SC, Creaser RA (2008). Cretaceous oceanic anoxic event 2 triggered by a massive magmatic episode. Nature.

[19] McAnena A (2013). Atlantic cooling associated with a marine biotic crisis during the mid-Cretaceous period. Nat. Geosci..

[20] Bodin S (2015). Large igneous provinces and organic carbon burial: Controls on global temperature and continental weathering during the Early Cretaceous. Global Planet. Change.

[21] Herrle JO (2015). Mid-Cretaceous High Arctic stratigraphy, climate, and oceanic anoxic events. Geology.

[22] Huber BT (2018). The rise and fall of the Cretaceous Hot Greenhouse climate. Global Planet. Change.

[23] Frey FA (2003). Leg 183 synthesis: Kerguelen Plateau-Broken Ridge-a large igneous province. Proc. Ocean Drill. Progr. Sci. Results..

[24] Huber BT (2011). Paleotemperature and paleosalinity inferences and chemostratigraphy across the Aptian/Albian boundary in the subtropical North Atlantic. Paleoceanography.

[25] Bralower TJ (1997). Mid-Cretaceous strontium-isotope stratigraphy of deep-sea sections. Geol. Soc. Am. Bull..

[26] Dias BB (2010). Modern seawater acidification: the response of foraminifera to high-CO_2_ conditions in the Mediterranean Sea. J. Geol. Soc..

[27] Kawahata H (2015). Linkage of deep sea rapid acidification process and extinction of benthic foraminifera in the deep sea at the Paleocene/Eocene transition. Island Arc.

[28] Pettit LR (2015). Seaweed fails to prevent ocean acidification impact on foraminifera along a shallow-water CO2 gradient. Ecol. Evol..

[29] Barker S, Elderfield H (2002). Foraminiferal calcification response to glacial-interglacial changes in atmospheric CO_2_. Science.

[30] Allison N (2010). Culture studies of the benthic foraminifera Elphidium williamsoni: Evaluating pH, ∆[CO32−] and inter-individual effects on test Mg/Ca. Chem. Geol..

[31] Davis CV (2017). Ocean acidification compromises a planktic calcifier with implications for global carbon cycling. Sci. Rep..

[32] Khanna N, Godbold JA, Austin WE, Paterson DM (2013). The impact of ocean acidification on the functional morphology of foraminifera. PLoS ONE.

[33] Bolton CT (2016). Decrease in coccolithophore calcification and CO_2_ since the middle Miocene. Nat. Commun..

[34] McClelland HLO (2016). Calcification response of a key phytoplankton family to millennial-scale environmental change. Sci. Rep..

[35] Henehan MJ (2017). Size-dependent response of foraminiferal calcification to seawater carbonate chemistry. Biogeosciences.

[36] Lirer F (2000). A new technique for retrieving calcareous microfossils from lithified lime deposits. Micropalaeontology.

[37] Huber BT (2016). Pforsams@microtax: A new online taxonomic database for planktonic foraminifera. Micropaleontology.

[38] Sigal J (1977). Essai de zonation du Crétacé méditerranéen à l’aide des foraminifères planctoniques. Géologie Méditerranéenne.

[39] Toyofuku T (2011). Mg/Ca and δ^18^O in the brackish shallow-water benthic foraminifer Ammonia ‘beccarii’. Mar. Micropaleontol..

[40] Ohkouchi N (2005). Biogeochemical processes in the saline meromictic Lake Kaiike, Japan: implications from molecular isotopic evidences of photosynthetic pigments. Environ. Microbiol..

[41] Selby D, Creaser RA (2003). Re–Os geochronology of organic rich sediments: an evaluation of organic matter analysis methods. Chem. Geol..

[42] Shirey SB, Walker RJ (1995). Carius tube digestion for low-blank rhenium-osmium analysis. Anal. Chem..

[43] Birck JL, Barman MR, Capmas F (1997). Re-Os isotopic measurements at the femtomole level in natural samples. Geostand. Newslett..

[44] Kuroda J (2010). Marine osmium isotope record across the Triassic–Jurassic boundary from a Pacific pelagic site. Geology.

[45] Nozaki T (2012). A method for rapid determination of Re and Os isotope compositions using ID-MC-ICP-MS combined with the sparging method. Geostand. Geoanal. Res..

[46] Smoliar MI, Walker RJ, Morgan JW (1996). Re-Os ages of group IIA, IIIA, IVA, and IVB iron meteorites. Science.

[47] Huang C (2010). Astronomical tuning of the Aptian Stage from Italian reference sections. Geology.

[48] Esser BK, Turekian KK (1993). The osmium isotopic composition of the continental crust. Geochim. Cosmochim. Acta.

[49] Allègre CJ, Luck JM (1980). Osmium isotopes as petrogenetic and geological tracers. Earth Planet. Sci. Lett..

[50] Müller RD (2008). Long-term sea-level fluctuations driven by ocean basin dynamics. Science.

[51] Reisberg L (1993). Os isotope systematics in ocean island basalts. Earth Planet. Sci. Lett..

[52] Yang HJ (1998). Petrogenesis of the flood basalts forming the northern Kerguelen Archipelago: Implications for the Kerguelen plume. J. Petrol..

