# Supplementary material for: Marine Os isotopic evidence for multiple volcanic episodes during Cretaceous Oceanic Anoxic Event 1b
Source: Sci Rep. 2020 Jul 28;10:12601. doi: 10.1038/s41598-020-69505-x (PMC7387342; doi:10.1038/s41598-020-69505-x)
Supplement: Supplementary file 1 — Supplementary file1 (PDF 2366 kb) [file 41598_2020_69505_MOESM1_ESM.pdf]

Supplementary Information for

"Marine Os isotopic evidence for multiple volcanic episodes during Cretaceous Oceanic Anoxic Event 1b"

by Hironao Matsumoto[1*] (matsumoto@aori.u-tokyo.ac.jp); Junichiro Kuroda[1]; Rodolfo Coccioni[2]; Fabrizio Frontalini[2]; Saburo Sakai[3]; Nanako O. Ogawa[3], Naohiko Ohkouchi[3]

[1]Atmosphere and Ocean Research Institute, The University of Tokyo, Japan

[2]DiSPeA, University of Urbino, Italy

[3]Japan Agency for Marine-Earth Science and Technology, Japan

Supplementary Figures

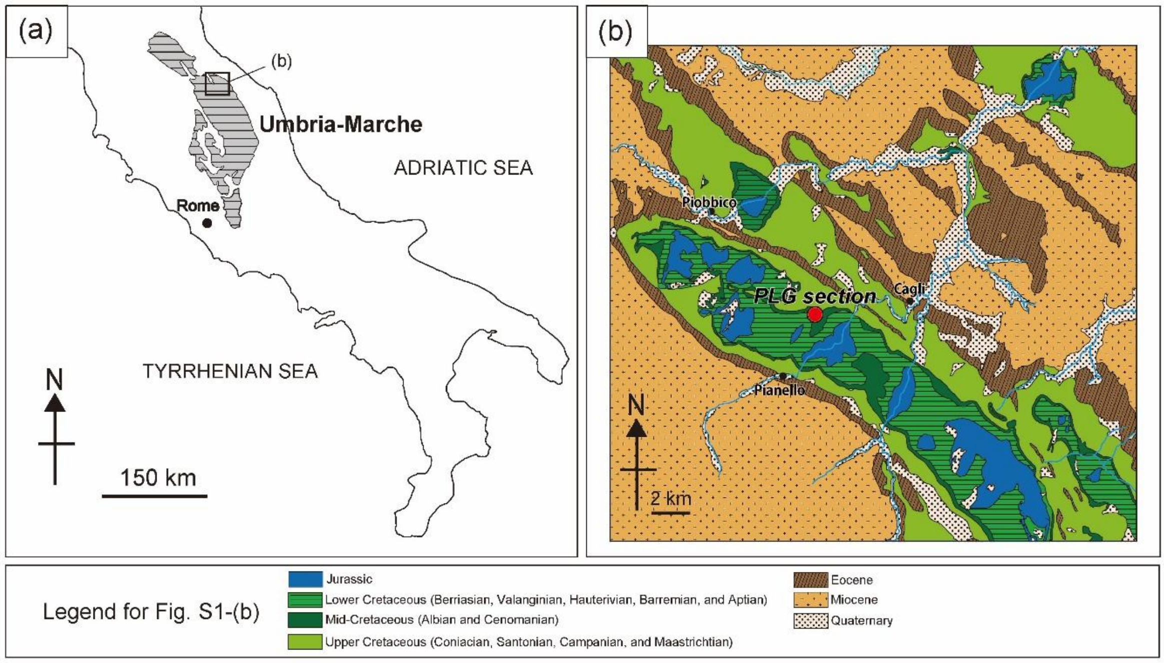

Supplementary Fig. S1: Geological map of the Umbria–Marche basin based on ref[53]. The map is created using Illustrator CS5.5 (https://www.adobe.com/products/illustrator.html).

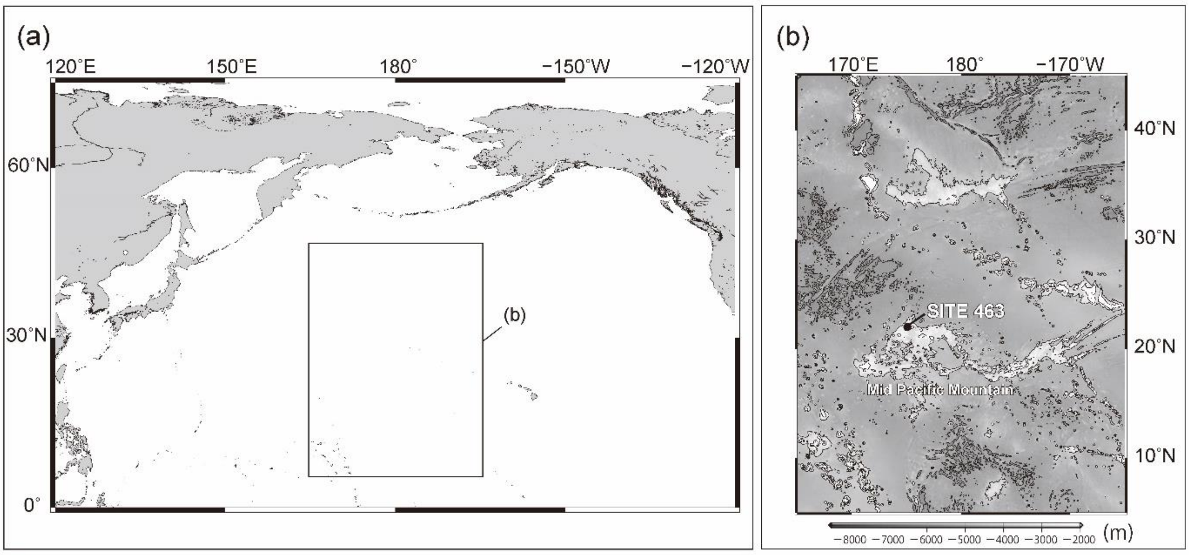

Supplementary Fig. S2: Drilling site of the DSDP Site 463. The map is generated using GMT (ver. 5.4.1) (Generic Mapping Tools: http://gmt.soest.hawaii.edu/home).

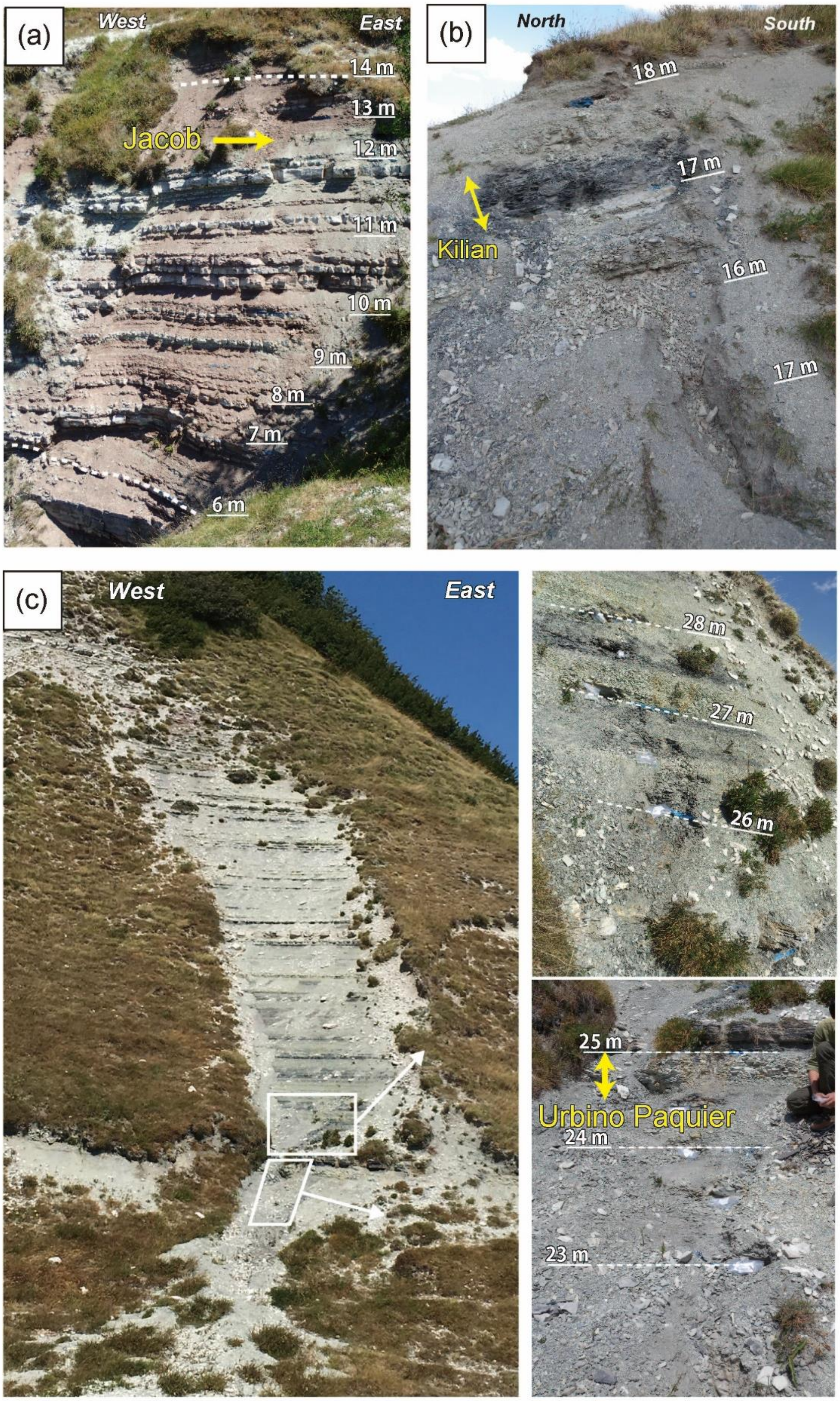

Supplementary Fig. S3: Outcrop images of the PLG section.

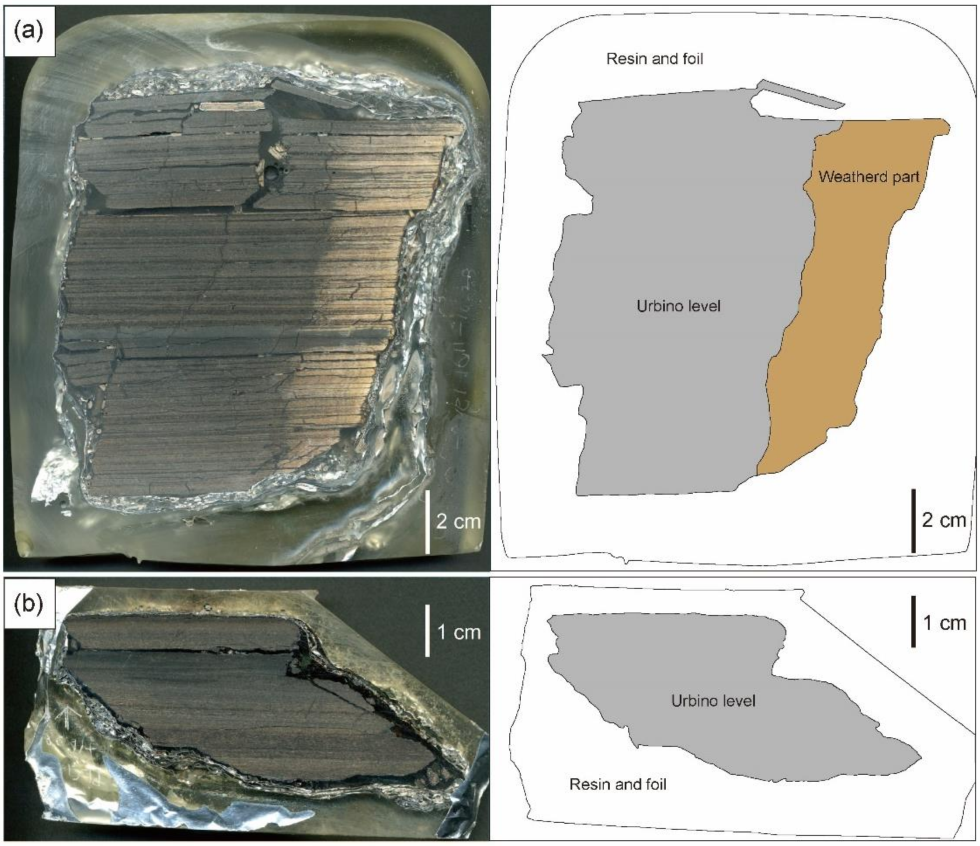

Supplementary Fig. S4: Slabs of the Urbino/Paquier equivalent horizon. (a) 25.11–25.13 m, (b) ~25.28 m. Brownish part in (a) is an apparently weathered part.

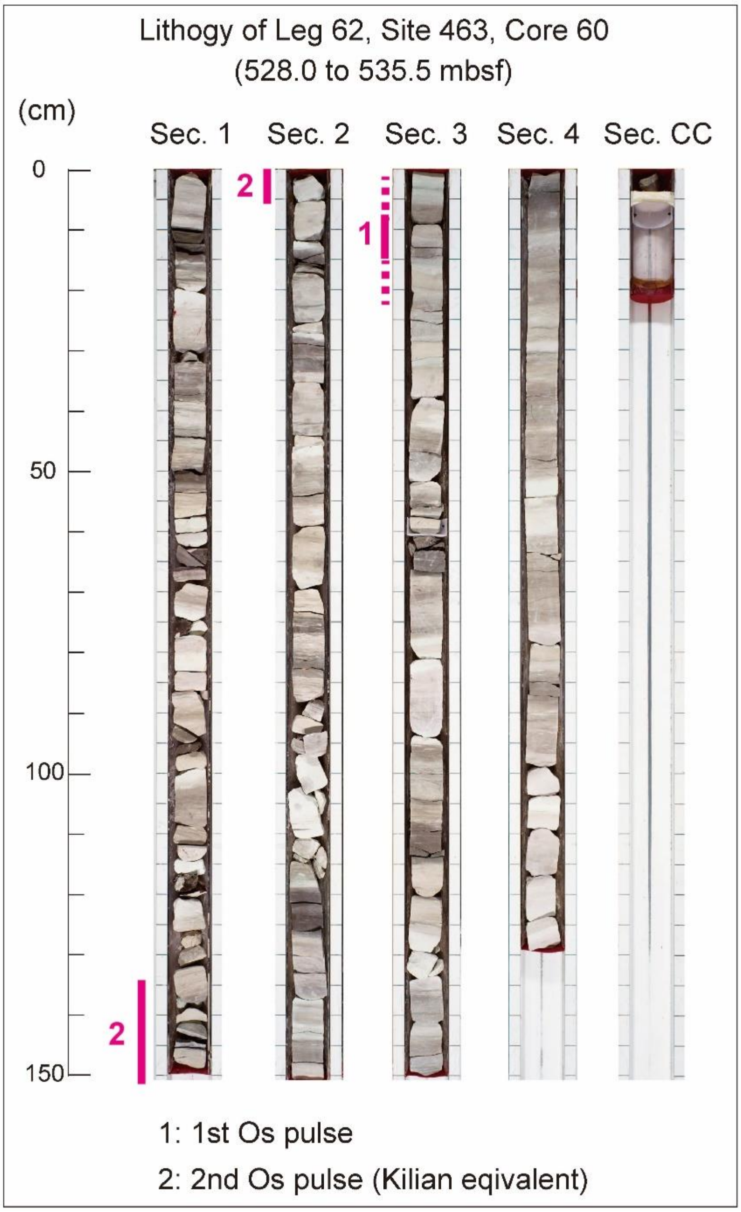

Supplementary Fig. S5. Lithological image of DSDP Site 463, core 60. The image is cited from http://deepseadrilling.org/cores/leg062/463.60R.PDF. Lithology consists of whitish limestone and brownish marlstone alternated with some chert layers.

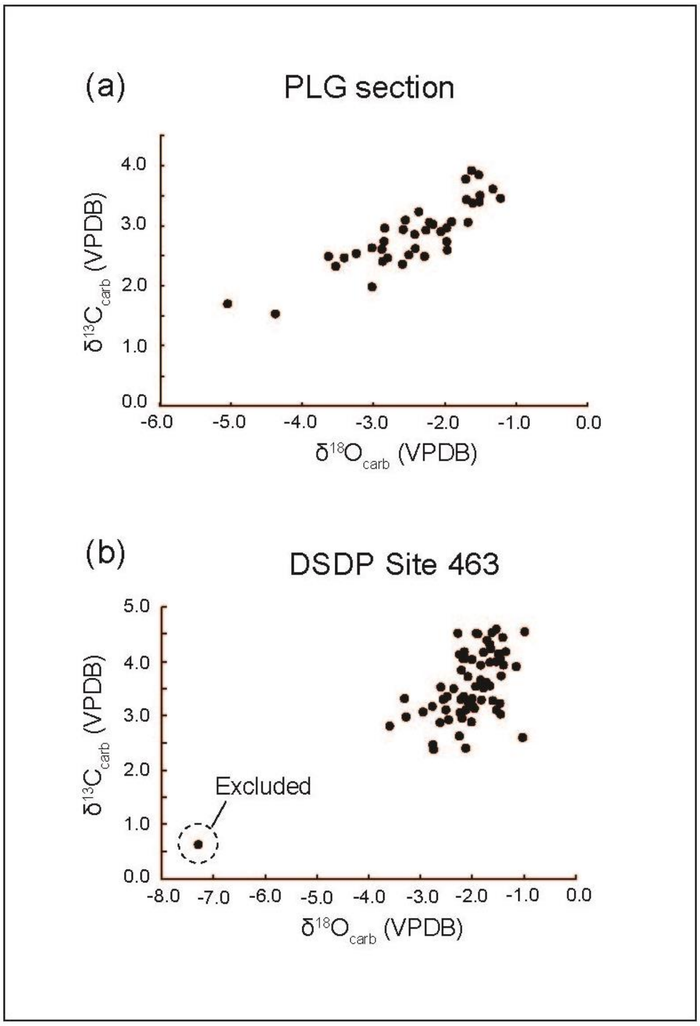

Supplementary Fig. S6: Cross plot of $\delta^{13}C_{carb}$ and $\delta^{18}O_{carb}$ of sedimentary rock samples from (a) the PLG section and (b) the DSDP Site 463.

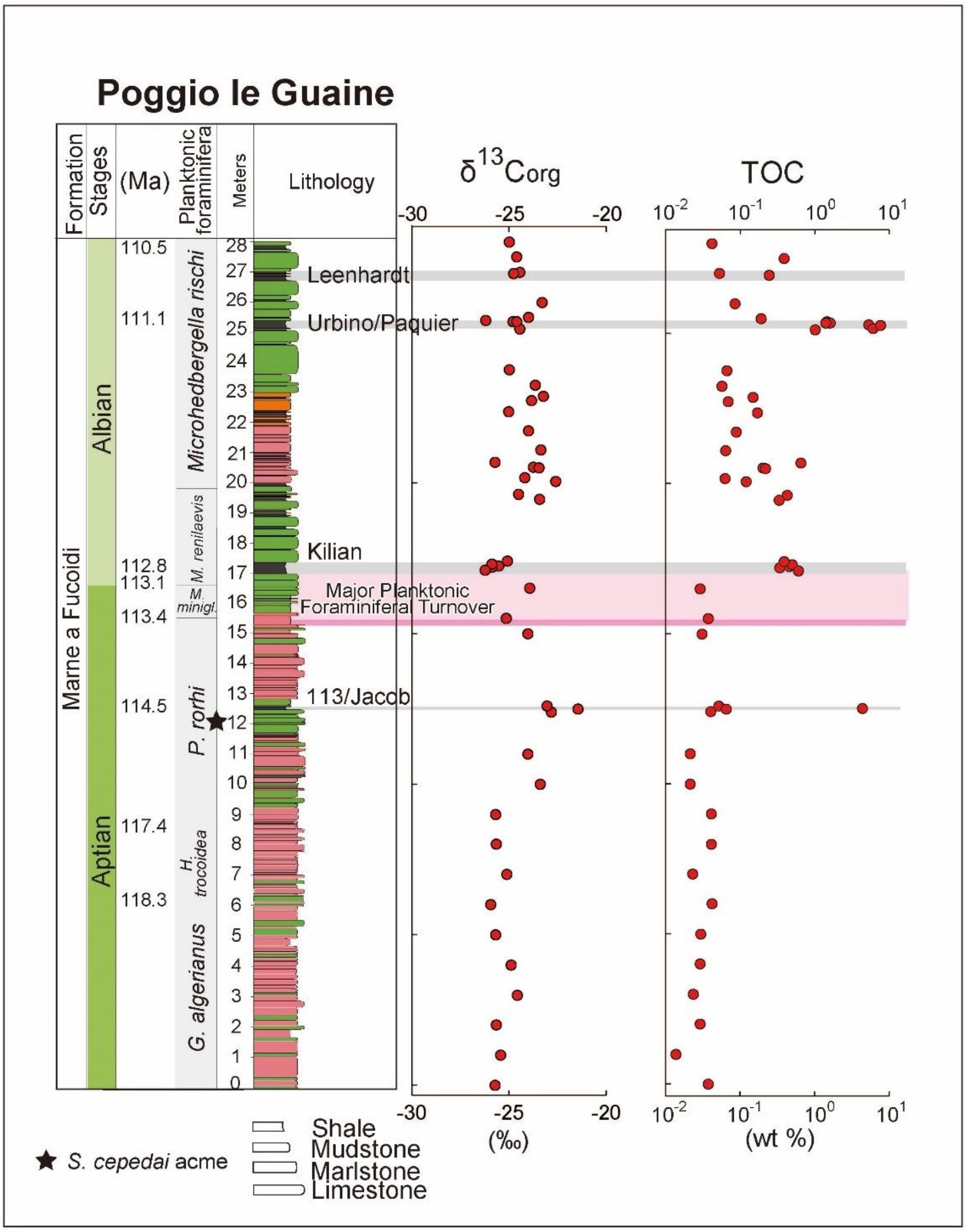

Supplementary Fig. S7: The $\delta^{13}C_{org}$ and TOC records at the PLG section.

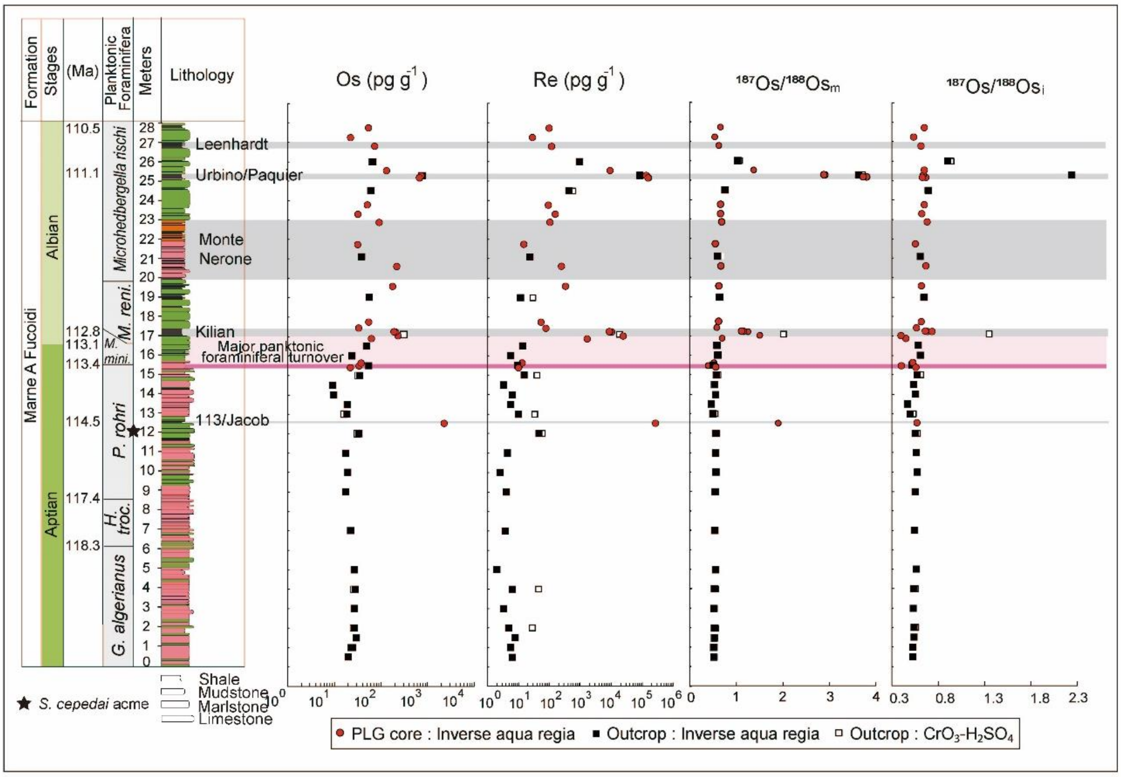

Supplementary Fig. S8: Os and Re concentration, $^{187}Os/^{188}Os_m$, and $^{187}Os/^{188}Os_i$ at the PLG section and core.

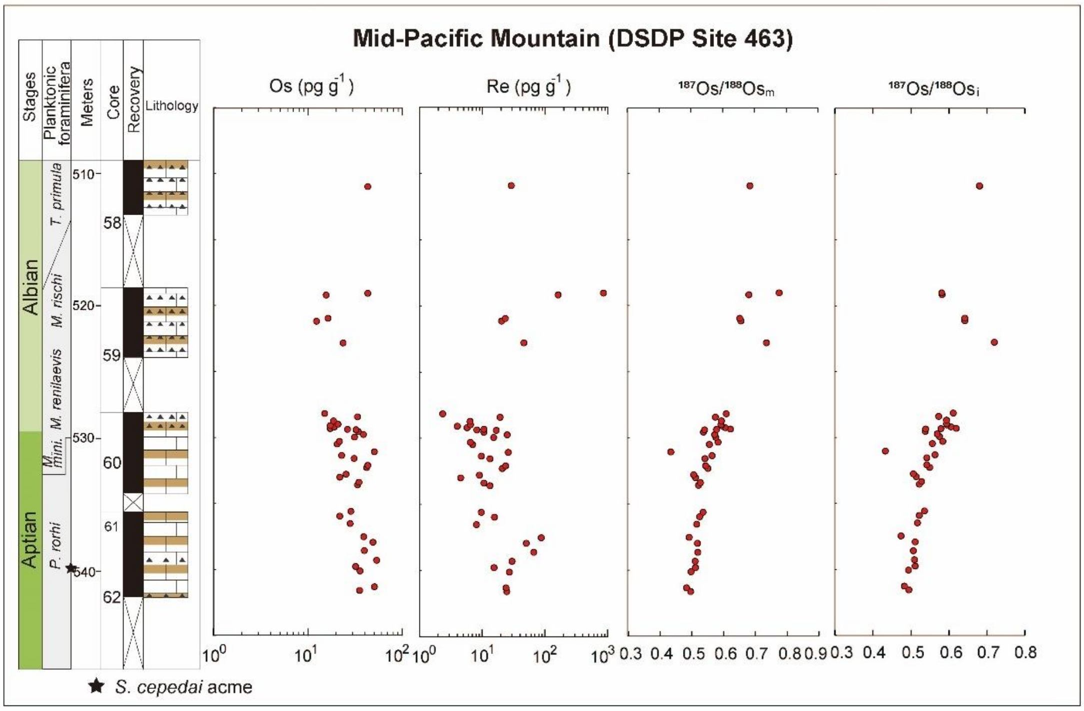

Supplementary Fig. S9: The Os and Re concentration, $^{187}Os/^{188}Os_m$, and $^{187}Os/^{188}Os_i$ at the DSDP Site 463.

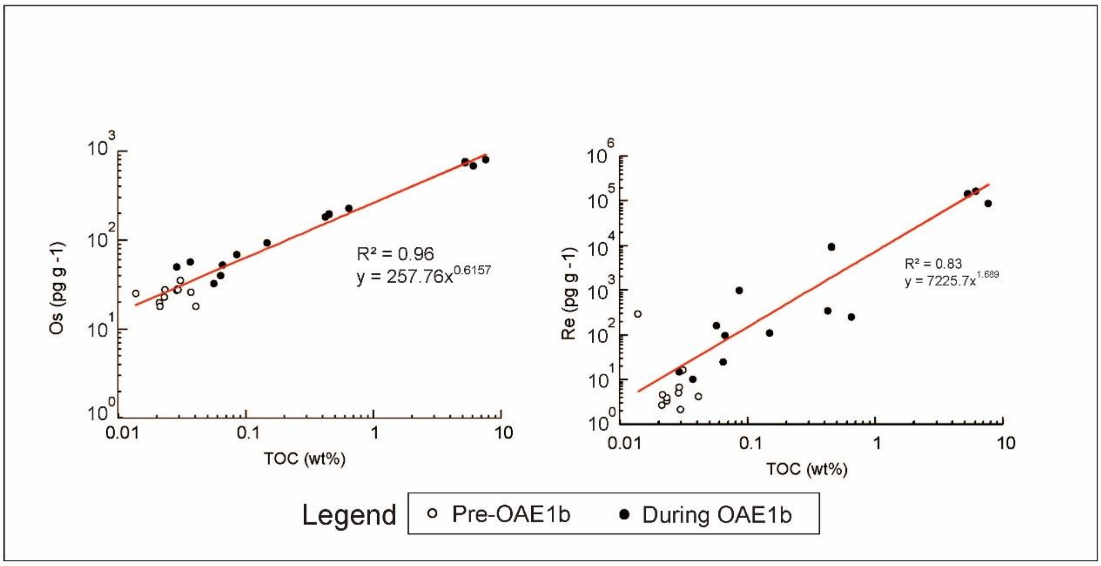

Supplementary Fig. S10: Cross plots of Os and Re concentrations against TOC of the PLG section. Black circles represent the sedimentary rock samples from OAE1b interval and white ones represent the sedimentary rock samples from the pre-OAE1b. Red lines show the approximate curves of plotted data containing both black and white circles.

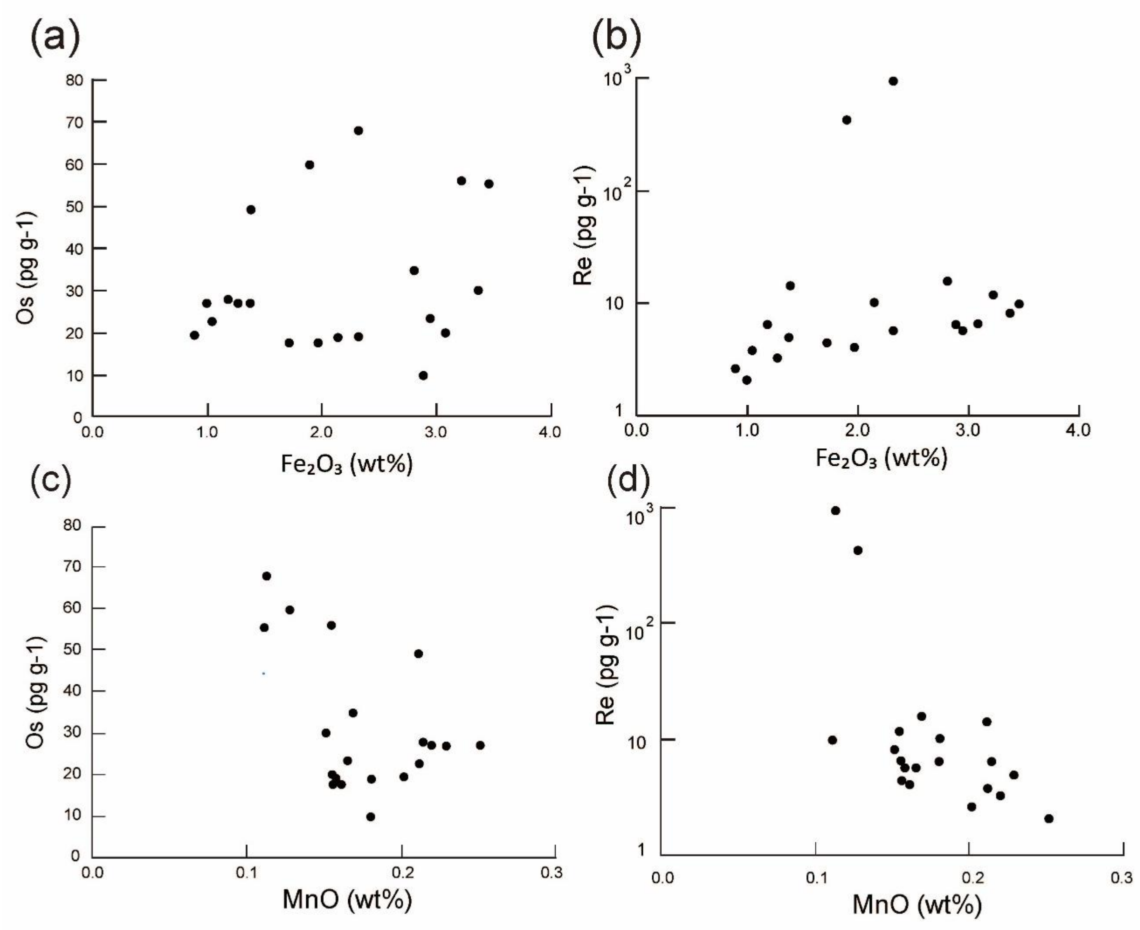

Supplementary Fig. S11: Cross plots of (a) Fe_2O_3 (wt%)–Os (pg g^{-1}), (b) Fe_2O_3 (wt%)–Re (pg g^{-1}), (c) MnO (wt%)–Os (pg g^{-1}), and (d) MnO (wt%)–Re (pg g^{-1}).

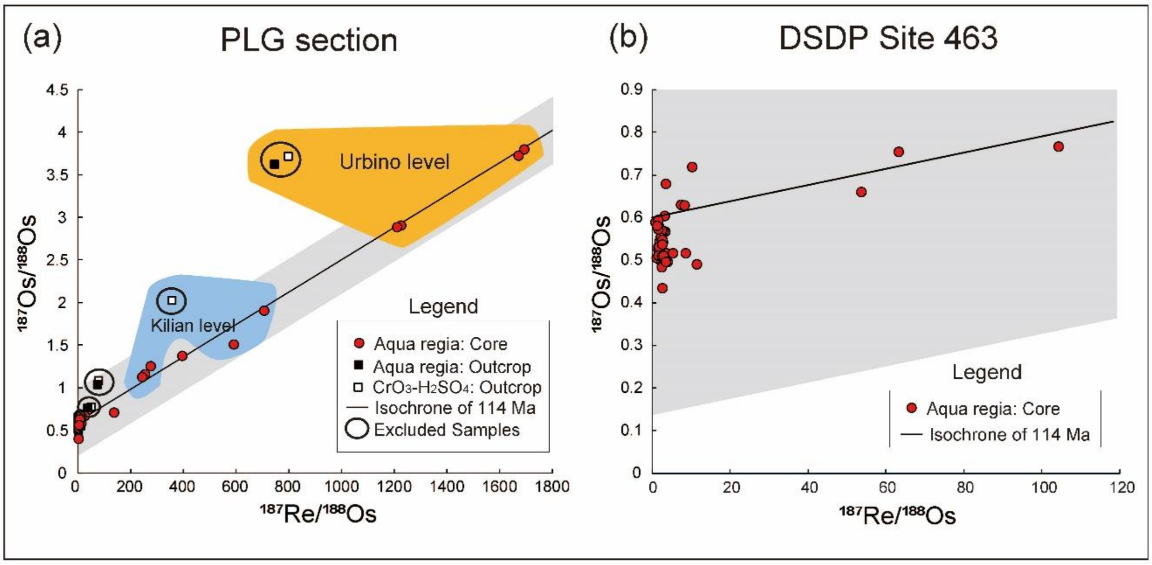

Supplementary Fig. S12: cross plot of $^{187}Re/^{188}Os$-$^{187}Os/^{188}Os_m$ of the PLG section and PLG core, and the DSDP Site463. Points in orange area represents the Urbino/Paquier equivalent level and points in blue area represents the Kilian equivalent level.

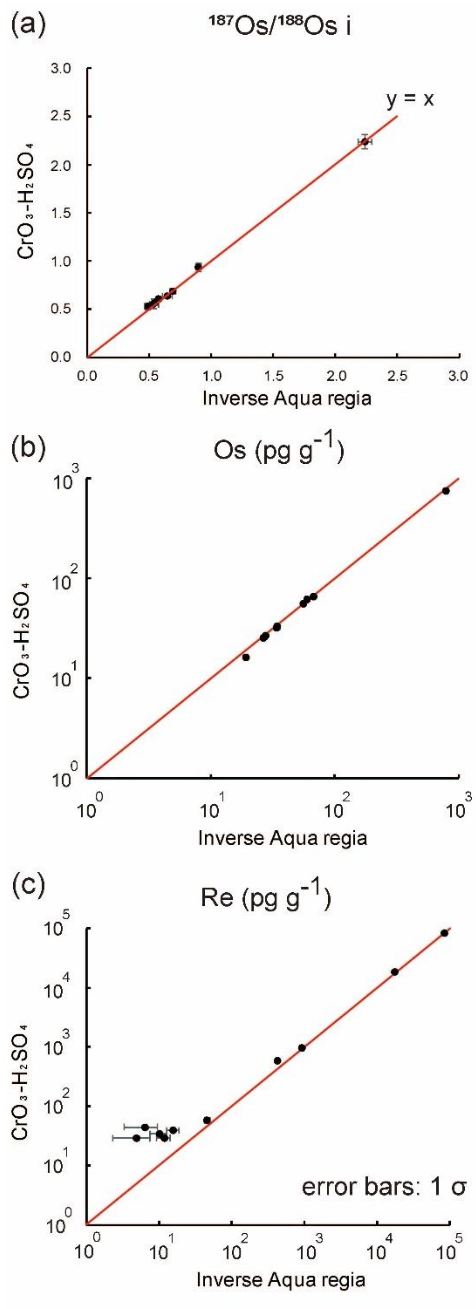

Supplementary Fig. S13: Comparison of $^{187}Os/^{188}Os_i$ and Os and Re concentrations of sedimentary rock samples of the PLG section treated with inverse aqua regia and CrO_3–H_2SO_4.

Supplementary Tables

Name (Date SampleID)	Depth (msl)	Date	$\delta^{13}C$ (‰VPDB)	1σ for $\delta^{13}C$	$\delta^{18}O$ (‰VPDB)	1σ for $\delta^{18}O$
171229 PLG-9.0	0	2017/12/29	3.024	0.005	-2.164	0.012
171229 PLG-8.5	0.5	2017/12/29	2.931	0.004	-2.269	0.006
171229 PLG-8.0	1	2017/12/29	2.862	0.004	-2.421	0.017
171229 PLG-7.5	1.5	2017/12/29	2.512	0.006	-2.499	0.009
171229 PLG-7.0	2	2017/12/29	2.598	0.004	-1.962	0.012
171229 PLG-6.0	3	2017/12/29	2.616	0.009	-2.415	0.012
171229 PLG-5.5	3.5	2017/12/29	2.740	0.004	-1.979	0.007
171229 PLG-4.5	4.5	2017/12/29	2.909	0.004	-2.051	0.012
171229 PLG-4.0	5	2017/12/29	2.488	0.005	-2.282	0.010
171229 PLG-3.5	5.5	2017/12/29	2.969	0.005	-1.978	0.009
171229 PLG-2.5	6.5	2017/12/29	3.508	0.007	-1.506	0.015
171229 PLG-1.35	7.65	2017/12/29	3.059	0.007	-2.210	0.009
171229 PLG-0.4	8.6	2017/12/29	3.459	0.005	-1.217	0.009
171229 PLG-0.5	9.5	2017/12/29	3.614	0.004	-1.325	0.013
171229 PLG+1.0	10	2017/12/29	3.402	0.008	-1.519	0.015
171229 PLG+1.5	10.5	2017/12/29	3.381	0.009	-1.604	0.011
171229 PLG+2.0	11	2017/12/29	3.775	0.004	-1.705	0.019
171229 PLG+2.5	11.5	2017/12/29	3.845	0.007	-1.530	0.014
171229 PLG+3.0	12	2017/12/29	3.921	0.006	-1.626	0.015
171230 PLG+3.6	12.6	2017/12/30	3.063	0.004	-1.674	0.010
171230 PLG+4.0	13	2017/12/30	3.436	0.003	-1.700	0.019
171230 PLG+5.0	14	2017/12/30	3.239	0.005	-2.363	0.012
171230 PLG+6.0	15	2017/12/30	2.940	0.011	-2.581	0.02
171230 PLG+7.0	16	2017/12/30	3.092	0.004	-2.551	0.011
171230 PLG+7.8	16.8	2017/12/30	2.959	0.007	-2.846	0.013
171230 PLG+8.55	17.55	2017/12/30	2.330	0.011	-3.534	0.015
171230 PLG+9.0	18	2017/12/30	2.544	0.005	-3.244	0.009
171230 PLG+10.0	19	2017/12/30	2.609	0.006	-2.886	0.009
171230 PLG+14.0	23	2017/12/30	2.464	0.008	-2.802	0.011
171230 PLG+150	24	2017/12/30	2.491	0.010	-3.627	0.017
171230 PLG+15.5	24.5	2017/12/30	2.735	0.003	-2.856	0.018
171230 PLG+16.6	25.6	2017/12/30	2.405	0.013	-2.877	0.03
171230 PLG+17.0	26	2017/12/30	2.470	0.006	-3.410	0.02
171230 PLG+17.5	26.5	2017/12/30	2.640	0.004	-3.023	0.015
171230 PLG+18.0	27	2017/12/30	1.535	0.007	-4.380	0.017
171230 PLG+19.0	28	2017/12/30	1.984	0.007	-3.027	0.02
171230 PLG+12.10	21.1	2017/12/30	2.363	0.012	-2.595	0.05
171230 PLG+13.0	22	2017/12/30	3.069	0.008	-1.907	0.03
171230 PLG+13.5	22.5	2017/12/30	1.707	0.017	-5.046	0.06

Supplementary Table S1: Stable carbon and oxygen isotopic composition of carbonate from the PLG section.

Name	Sample ID	Depth	Date	$\delta^{13}C$	1σ for $\delta^{13}C$	$\delta^{18}O$	1σ for $\delta^{13}C$	Remarks
(Date Site_Core-Section_Top(cm)-Bottom(cm))		(mbsf)		(‰VPDB)		(‰VPDB)		
181001 463_56-1_30-34	463-2-56-1-2	490.3	2018/10/1	2.404	0.011	-2.134	0.02	
181001 463_57-2_70-72	463-2-57-2-3	500.2	2018/10/1	2.470	0.007	-2.766	0.011	
180404 463_58-1_46-48	463-1-58-1-1	509.46	2018/4/4	2.922	0.005	-2.462	0.015	
181001 463_58-1_131-135	463-2-58_1_4	510.31	2018/10/1	2.624	0.015	-2.252	0.017	
180404 463_58-2_44-50	463-1-58-2-1	510.94	2018/4/4	3.025	0.002	-1.453	0.007	
180919 463_58-2_83-86	463-1-58-2-2	511.33	2018/9/19	2.596	0.007	-1.031	0.013	
180404 463_58-3_40-42	463-1-58-3-2	512.36	2018/4/4	2.881	0.005	-2.027	0.011	
181001 463_58-3_107_111	463-1-58-3-3	513.07	2018/10/1	2.375	0.010	-2.748	0.012	
180404 463_59-1_14_18	463-1-59-1-1	518.64	2018/4/4	3.299	0.003	-2.574	0.009	
180404 463_59-1_36_38	463-1-59-1-2	518.86	2018/4/4	3.227	0.004	-2.073	0.013	
180404 463_59-1_54_56	463-1-59-1-3	519.04	2018/4/4	3.311	0.002	-2.008	0.007	
180422 463_59-1_68_71(2)	463-1-59-1-4	519.18	2018/4/22	3.300	0.007	-2.558	0.009	
180422 463_59-1_83-86(2)	463-1-59-1-5	519.33	2018/4/22	3.315	0.004	-3.313	0.016	
180404 463_59-1_115-119	463-1-59-1-6	519.65	2018/4/4	3.109	0.003	-2.136	0.003	
180404 463_59-2_6-9	463-1-59-2-1	520.06	2018/4/4	3.177	0.004	-2.777	0.008	
180404 463_59-2_21-26	463-1-59-2-2	520.21	2018/4/4	3.225	0.005	-1.466	0.009	
180422 463_59-2_44-48(2)	463-1-59-2-3	520.44	2018/4/22	2.948	0.007	-2.203	0.008	
180404 463_59-2_74-78	463-1-59-2-4	520.74	2018/4/4	3.057	0.004	-2.241	0.006	
180404 463_59-2_96-98	463-1-59-2-5	520.96	2018/4/4	2.976	0.003	-3.276	0.010	
180404 463_59-2_116-118	463-1-59-2-6	521.16	2018/4/4	3.071	0.0018	-2.954	0.006	
180404 463_59-2_135-137	463-1-59-2-7	521.35	2018/4/4	3.177	0.003	-1.992	0.009	
180422 463_59-3_6-9(2)	463-1-59-3-1	521.56	2018/4/22	3.104	0.006	-2.511	0.008	
180422 463_59-3_25-27(2)	463-1-59-3-2	521.75	2018/4/22	2.874	0.002	-2.626	0.007	
180822 463_59-3_95-97(2)	463-1-59-3-3	522.45	2018/8/22	3.361	0.003	-2.168	0.008	
180404 463_59-3_118-120	463-1-59-3-4	522.68	2018/4/4	3.510	0.006	-1.784	0.004	
180404 463_59-3_133-135	463-1-59-3-5	522.83	2018/4/4	3.309	0.002	-2.223	0.013	
180404 463_59-4_7-11	463-1-59-4-1	523.07	2018/4/4	0.631	0.004	-7.283	0.007	Excluded from the discussion
180822 463_59-4_7-11(2)	463-1-59-4-1	523.07	2018/8/22	3.110	0.004	-1.536	0.007	
180404 463_59-CC_10-13	463-1-59-CC-1	523.4	2018/4/4	3.280	0.003	-1.615	0.009	
180404 463_60-1_18-20	463-1-60-1-1	528.18	2018/4/4	3.647	0.002	-1.842	0.007	
180822 463_60-1_20-22(2)	463-1-60-1-2	528.2	2018/8/22	3.551	0.004	-1.659	0.007	
180404 463_60-1_44-46	463-1-60-1-3	528.44	2018/4/4	3.715	0.005	-2.086	0.012	
180404 463_60-1_73-75	463-1-60-1-4	528.73	2018/4/4	3.613	0.003	-1.734	0.007	
180822 463_60-1_94-99(2)	463-1-60-1-5	528.94	2018/8/22	3.296	0.003	-1.825	0.012	
180404 463_60-1_100-102	463-1-60-1-6	529	2018/4/4	3.355	0.003	-2.489	0.005	
180919 463_60-1_122-127	463-2-60-1-2	529.22	2018/9/19	3.129	0.006	-1.969	0.012	
180404 463_60-1_133-138	463-2-60-1-7	529.33	2018/4/4	2.806	0.004	-3.594	0.011	
180919 463_60-1_138-141	463-2-60-1-3	529.38	2018/9/19	3.539	0.005	-1.936	0.010	
180406 463_60-2_6-8	463-1-60-2-1	529.56	2018/4/6	3.521	0.003	-2.611	0.009	
180406 463_60-2_27-29	463-1-60-2-2	529.77	2018/4/6	3.734	0.004	-1.449	0.007	
180406 463_60-2_82-85	463-1-60-2-5	530.32	2018/4/6	3.650	0.007	-1.848	0.015	
180406 463_60-2_100-103	463-1-60-2-6	530.5	2018/4/6	3.497	0.005	-2.368	0.012	
180822 463_60-2_136-138(2)	463-1-60-2-7	530.86	2018/8/22	3.839	0.005	-2.219	0.009	
180822 463_60-3_8-12(2)	463-1-60-3-1	531.08	2018/8/22	3.980	0.006	-1.662	0.007	
180406 463_60-3_36-40	463-1-60-3-2	531.36	2018/4/6	4.051	0.003	-2.140	0.010	
180406 463_60-3_58-59	463-1-60-3-3	531.58	2018/4/6	4.043	0.003	-2.187	0.014	
180919 463_60-3_80-82	463-2-60-3-1	531.8	2018/9/19	3.936	0.003	-1.839	0.010	
180919 463_60-3_111-117	463-2-60-3-2	532.11	2018/9/19	3.907	0.007	-1.152	0.017	
180919 463_60-3_130-134	463-2-60-3-3	532.3	2018/9/19	4.028	0.007	-1.458	0.010	
180919 463_60-4_10-13	463-2-60-4-1	532.6	2018/9/19	4.391	0.006	-1.718	0.010	
180919 463_60-4_30-32	463-2-60-4-2	532.8	2018/9/19	4.173	0.005	-2.156	0.009	
181001 463_60-4_52-54	463-2-60-4-3	533.02	2018/10/1	4.093	0.004	-2.163	0.009	
180919 463_60-4_77-79	463-2-60-4-4	533.27	2018/9/19	4.232	0.008	-1.657	0.011	
180919 463_60-4_110-117	463-2-60-4-6	533.6	2018/9/19	4.177	0.009	-1.358	0.008	
180919 463_61-1_12-15	463-2-61-1-1	535.62	2018/9/19	4.001	0.008	-1.547	0.009	
180919 463_61-1_70-72	463-2-61-1-2	536.2	2018/9/19	3.927	0.009	-1.399	0.006	
180919 463_61-1_103-105	463-2-61-1-3	536.53	2018/9/19	4.028	0.007	-2.004	0.011	
180919 463_62-1_3-5	463-2-62-1-1	537.53	2018/9/19	4.138	0.010	-1.506	0.015	
180822 463_62-1_47-49(2)	463-1-62-1-1	537.97	2018/8/22	4.168	0.005	-1.792	0.008	
180822 463_62-1_114-116	463-2-62-1-2	538.64	2018/8/22	4.119	0.007	-2.255	0.007	
180822 463_62-2_5-7	463-2-62-2-1	539.05	2018/8/22	4.514	0.002	-2.281	0.008	
180822 463_62-2_32-35	463-2-62-2-2	539.32	2018/8/22	4.521	0.004	-1.918	0.004	
180822 463_62-2_80-81	463-2-62-2-3	539.8	2018/8/22	4.505	0.006	-1.901	0.005	
180822 463_62-2_113-115	463-2-62-2-4	540.13	2018/8/22	4.589	0.009	-1.546	0.01	
180822 463_62-3_2-9	463-2-62-3-1	540.52	2018/8/22	4.543	0.005	-0.988	0.007	
180822 463_62-3_35-42	463-2-62-3-2	540.85	2018/8/22	4.529	0.007	-1.620	0.007	
180822 463_62-3_83-88	463-2-62-3-3	541.33	2018/8/22	4.312	0.008	-1.658	0.013	
180822 463_62-3_111-113	463-2-62-3-4	541.61	2018/8/22	4.438	0.005	-1.424	0.007	

Supplementary Table S2: Stable carbon and oxygen isotopic composition of carbonate from DSDP Site 463.

Sample ID	Lab ID	Core or Outcrop	lithology	Depth	Date	$\delta^{13}C$	C wt.% (TOC)
				(msl)		(‰VPDB)	(wt%)
PLG-9	HM-C-16	Outcrop	marlstone	0	2018/7/23	-25.7	0.04
PLG-8	HM-C-17	Outcrop	marlstone	1	2018/7/12	-25.4	0.01
PLG-7	HM-C-18	Outcrop	marlstone	2	2018/7/12	-25.7	0.03
PLG-6	HM-C-19	Outcrop	marlstone	3	2018/7/12	-24.6	0.02
PLG-5	HM-C-20	Outcrop	marlstone	4	2018/7/12	-24.9	0.03
PLG-4	HM-C-21	Outcrop	marlstone	5	2018/7/23	-25.7	0.03
PLG-3	HM-C-22	Outcrop	marlstone	6	2018/7/12	-26.0	0.04
PLG-2	HM-C-23	Outcrop	marlstone	7	2018/7/12	-25.1	0.02
PLG-1	HM-C-24	Outcrop	marlstone	8	2018/7/12	-25.7	0.04
PLG0	HM-C-25	Outcrop	marlstone	9	2018/7/12	-25.7	0.04
PLG+1.0	HM-C-26	Outcrop	marlstone	10	2018/7/12	-23.4	0.02
PLG+2.0	HM-C-27	Outcrop	marlstone	11	2018/7/12	-24.1	0.02
PLG+3.4	HM-C-28	Outcrop	marlstone	12.4	2018/7/12	-22.8	0.04
PLG+3.5①	HM-C-29	Outcrop	black shale	12.5	2018/7/12	-21.5	0.06
PLG+3.5③	HM-C-30-1	Outcrop	black shale	12.52	2018/7/23	Too large	4.4
PLG+3.5③	HM-C-30-2	Outcrop	black shale	12.52	2018/8/8-7	-19.4	6.1
PLG+3.5③	HM-C-30-3	Outcrop	black shale	12.52	2018/8/8-7	-19.1	5.1
PLG+3.5③	HM-C-30-4	Outcrop	black shale	12.52	2018/8/8-7	-18.8	5.2
PLG+3.6	HM-C-31	Outcrop	marlstone	12.6	2018/7/23	-23.1	0.05
PLG+6.0	HM-C-32	Outcrop	marlstone	15	2018/7/23	-24.0	0.03
PLG+6.5	HM-C-33	Outcrop	marlstone	15.5	2018/7/23	-25.2	0.04
PLG+7.5	HM-C-08	Outcrop	marlstone	16.5	2018/7/23	-23.9	0.03
PLG+8.1	HM-C-34	Outcrop	black shale	17.1	2018/7/23	-26.3	0.60
PLG+8.2	HM-C-35	Outcrop	black shale	17.2	2018/7/23	-25.9	0.34
PLG-C_64.49	HM-C-15	Core	black shale	17.24	2018/7/23	-25.6	0.45
PLG+8.3	HM-C-36	Outcrop	black shale	17.3	2018/7/23	-25.9	0.49
PLG+8.4	HM-C-37	Outcrop	black shale	17.4	2018/7/23	-25.1	0.39
PLG+10.45	HM-C-49	Outcrop	black shale	19.45	2018/7/23	-23.5	0.33
PLG-C62.11-62.2	HM-C-14	Core	marlstone	19.62	2018/7/23	-24.5	0.42
PLG-C61.67-61.73	HM-C-13	Core	black shale	20.06	2018/7/23	-22.6	0.12
PLG_C61.56-61.59	HM-C-12	Core	marlstone	20.17	2018/7/12	-24.2	0.06
PLG+11.5	HM-C-50	Outcrop	mudstone	20.5	2018/7/23	-23.5	0.22
PLG-C_61.2	HM-C-11	Core	black shale	20.53	2018/7/23	-23.8	0.20
PLG-C_61.05-61.2	HM-C-10	Core	black shale	20.68	2018/7/23	-25.7	0.65
PLG+12.10	HM-C-48	Outcrop	mudstone	21.1	2018/7/23	-23.4	0.06
PLG-C_60.00-60.15	HM-C-09	Core	marlstone	21.73	2018/7/12	-24.0	0.09
PLG-C_59.37-59.38	HM-C-07	Core	marlstone	22.36	2018/7/23	-25.0	0.17
PLG-C_58.99	HM-C-06	Core	marlstone	22.74	2018/7/23	-23.9	0.07
PLG-C_58.86-58.87	HM-C-05	Core	black shale	22.87	2018/7/12	-23.3	0.15
PLG-C_58.48-58.40	HM-C-04	Core	marlstone	23.25	2018/7/12	-23.7	0.06
PLG-C_57.97	HM-C-03	Core	marlstone	23.76	2018/7/23	-25.0	0.07
PLG+16.12	HM-C-38	Outcrop	black shale	25.12	2018/7/23	-24.5	1.0
PLG-C_56.56-56.58	HM-C-02-1	Core	black shale	25.17	2018/7/23	Too large	6.1
PLG-C_56.56-56.58	HM-C-02-2	Core	black shale	25.17	2018/8/8-7	-23.5	8.1
PLG-C_56.56-56.58	HM-C-02-3	Core	black shale	25.17	2018/8/8-7	-23.3	7.5
PLG-C_56.56-56.58	HM-C-02-4	Core	black shale	25.17	2018/8/8-7	-24.6	7.0
PLG+16.28	HM-C-39-1	Outcrop	black shale	25.28	2018/7/23	Too large	7.7
PLG+16.28	HM-C-39-2	Outcrop	black shale	25.28	2018/8/8-7	-23.2	5.7
PLG+16.28	HM-C-39-3	Outcrop	black shale	25.28	2018/8/8-7	-23.0	5.2
PLG+16.28	HM-C-39-4	Outcrop	black shale	25.28	2018/8/8-7	-23.1	6.7
PLG-C_56.44-56.48	HM-C-01-1	Core	black shale	25.29	2018/7/23	Too large	5.3
PLG-C_56.44-56.48	HM-C-01-2	Core	black shale	25.29	2018/8/8-7	-23.6	5.7
PLG-C_56.44-56.48	HM-C-01-3	Core	black shale	25.29	2018/8/8-7	-22.5	5.8
PLG-C_56.44-56.48	HM-C-01-4	Core	black shale	25.29	2018/8/8-7	-23.0	4.6
PLG+16.35	HM-C-40-1	Outcrop	black shale	25.35	2018/7/12	Too small	-
PLG+16.35	HM-C-40-3	Outcrop	black shale	25.35	2018/7/23	-24.8	1.6
PLG+16.35	HM-C-40-2	Outcrop	black shale	25.35	2018/7/12	-24.6	1.4
PLG+16.4	HM-C-42	Outcrop	black shale	25.4	2018/7/23	-26.2	1.5
PLG+16.5	HM-C-41	Outcrop	black shale	25.5	2018/7/23	-24.0	0.19
PLG+17	HM-C-44	Outcrop	black shale	26	2018/7/23	-23.3	0.09
PLG+17.95	HM-C-45	Outcrop	marlstone	26.95	2018/7/23	-24.8	0.25
PLG+18	HM-C-46	Outcrop	marlstone	27	2018/7/23	-24.5	0.05
PLG+18.5	HM-C-47	Outcrop	black shale	27.5	2018/7/23	-24.6	0.39
PLG+19	HM-C-43	Outcrop	marlstone	28	2018/7/23	-25.0	0.04

Supplementary Table S3: Total organic carbon content and stable carbon isotopic composition of organic matter at the PLG section and core.

Sample ID	Lab ID	Depth	$\delta^{13}C$	C wt.% (TOC)
		(mbsf)	(‰VPDB)	(wt%)
463_58-1_46-48	HM 1	509.46	-26.5	0.06
463_59-1_14-18	HM 2	518.64	-27.0	0.04
463_59-3_95-97	HM 3	522.45	-26.5	0.07
463_59-4_7-11	HM 4	523.07	-27.5	0.12
463_59-CC_10-13	HM 5	523.4	-26.6	0.02
463_60-2_82-85	HM 6	530.32	-27.0	0.51
463_60-2_27-29	HM 7	529.77	-27.1	0.10
463_60-3_8-12	HM 8	531.08	-27.0	0.12
463_60-4_90-93	HM 9	531.08	-27.1	0.08
463_60-4_110-117	HM 10	533.6	-27.6	0.10
463_61-1_12-15	HM 11	535.62	-27.0	0.05
462_61-1_103-105	HM 12	536.53	-27.0	0.09
463_62-2_113-115	HM 13	540.13	-27.1	0.07
463_62-3_83-88	HM 14	541.33	-27.0	0.07
463_62-3_111-113	HM 15	541.98	-27.4	0.07

Supplementary Table S4: Total organic carbon content and stable carbon isotopic composition of organic matter at the DSDP Site 463.

Sample Name	Lithology	Depth (msl)	Age (Ma)	Digestion liquid	Core/ outcrop	Os conc (pg g-1)	1SD	1SE	187Os/ 188Osm	1SD	1SE	Re conc (pg g-1)	1SD	1SE	187Re/ 188Os	1SD	1SE	187Os/ 188Osi	1SD	1SE
PLG-8.5	Marlsrtone	0.5	119	In. aqua regia	Outcrop	19.98	0.3	0.05	0.526	0.018	0.005	6.5.E+00	1.3.E+00	3.E-01	1.64	0.3	0.07	0.522	0.018	0.005
PLG-8	Marlsrtone	1	119	In. aqua regia	Outcrop	23.37	0.2	0.03	0.526	0.009	0.003	5.7.E+00	1.1.E+00	3.E-01	1.22	0.2	0.06	0.523	0.009	0.003
PLG-7.5	Marlsrtone	1.5	119	In. aqua regia	Outcrop	29.95	0.2	0.04	0.540	0.011	0.003	8.1.E+00	1.3.E+00	3.E-01	1.37	0.2	0.05	0.537	0.011	0.003
PLG-7.0	Marlsrtone	2	120	CrO_3-H_2SO_4	Outcrop	25.57	0.5	0.08	0.567	0.03	0.011	2.88.E+01	3.E+00	6.E-01	5.7	0.5	0.12	0.56	0.03	0.011
PLG-7.0	Marlsrtone	2	120	In. aqua regia	Outcrop	26.9	0.9	0.14	0.540	0.05	0.016	4.9.E+00	1.7.E+00	4.E-01	0.9	0.3	0.07	0.54	0.05	0.016
PLG-6.0	Marlsrtone	3	119	In. aqua regia	Outcrop	27.02	0.5	0.07	0.529	0.03	0.008	3.3.E+00	1.7.E+00	4.E-01	0.61	0.3	0.07	0.528	0.03	0.008
PLG-5.0	Marlsrtone	4	120	CrO_3-H_2SO_4	Outcrop	26.61	0.3	0.05	0.566	0.009	0.003	4.47.E+01	3.E+00	7.E-01	8.6	0.6	0.13	0.55	0.009	0.003
PLG-5.0	Marlsrtone	4	120	In. aqua regia	Outcrop	27.84	0.3	0.05	0.534	0.011	0.004	6.4.E+00	1.8.E+00	4.E-01	1.2	0.3	0.07	0.53	0.011	0.004
PLG-4.0	Marlsrtone	5	119	In. aqua regia	Outcrop	27.02	0.3	0.05	0.566	0.02	0.007	2.1.E+00	1.7.E+00	4.E-01	0.39	0.3	0.07	0.565	0.02	0.007
PLG-2.0	Marlsrtone	7	117	In. aqua regia	Outcrop	22.59	0.3	0.05	0.542	0.017	0.005	3.8.E+00	1.8.E+00	4.E-01	0.85	0.4	0.09	0.541	0.017	0.005
PLG0.0	Marlsrtone	9	117	In. aqua regia	Outcrop	17.6	0.2	0.03	0.554	0.015	0.005	4.0.E+00	1.8.E+00	4.E-01	1.17	0.5	0.11	0.551	0.016	0.005
PLG+1.0	Marlsrtone	10	115	In. aqua regia	Outcrop	19.38	0.13	0.02	0.573	0.011	0.003	2.6.E+00	1.7.E+00	4.E-01	0.68	0.5	0.10	0.571	0.011	0.003
PLG+2.0	Marlsrtone	11	115	In. aqua regia	Outcrop	17.57	0.16	0.03	0.566	0.016	0.005	4.4.E+00	1.8.E+00	4.E-01	1.28	0.5	0.11	0.564	0.016	0.005
PLG+3.0	Limestone	12	115	CrO_3-H_2SO_4	Outcrop	31.78	0.4	0.06	0.591	0.012	0.004	5.83.E+01	3.E+00	8.E-01	9.4	0.6	0.13	0.57	0.012	0.004
PLG+3.0	Limestone	12	115	In. aqua regia	Outcrop	34.2	0.5	0.08	0.564	0.03	0.009	4.61.E+01	2.E+00	5.E-01	6.8	0.4	0.08	0.55	0.03	0.009
PLG-C-69.20	Black shale (Jacob)	12.53	114	In. aqua regia	Core	2275	43	7	1.908	0.04	0.012	2.70.E+05	1.E+04	2.E+03	705	28	6	0.567	0.07	0.016
PLG+4.0	Marlsrtone	13	114	CrO_3-H_2SO_4	Outcrop	16.24	0.4	0.08	0.550	0.03	0.009	3.44.E+01	3.E+00	6.E-01	10.8	0.8	0.18	0.53	0.03	0.009
PLG+4.0	Marlsrtone	13	114	In. aqua regia	Outcrop	18.9	0.3	0.09	0.50	0.02	0.014	1.01.E+01	1.8.E+00	4.E-01	2.7	0.5	0.11	0.495	0.02	0.014
PLG+4.5	Marlsrtone	13.5	114	In. aqua regia	Outcrop	19.03	0.10	0.015	0.471	0.005	0.002	5.7.E+00	1.6.E+00	4.E-01	1.50	0.4	0.10	0.469	0.005	0.002
PLG+5	Limestone	14	114	In. aqua regia	Outcrop	9.75	0.14	0.02	0.561	0.018	0.005	6.4.E+00	1.3.E+00	3.E-01	3.33	0.7	0.16	0.555	0.018	0.005
PLG+5.5	Marlstone	14.5	114	In. aqua regia	Outcrop	9.30	0.11	0.017	0.537	0.015	0.005	3.2.E+00	1.7.E+00	4.E-01	1.8	0.9	0.2	0.534	0.015	0.005
PLG+6.0	Marlstone	15	113	CrO_3-H_2SO_4	Outcrop	32.94	0.4	0.06	0.617	0.02	0.005	3.95.E+01	3.E+00	7.E-01	6.1	0.5	0.11	0.61	0.016	0.005
PLG+6.0	Marlstone	15	113	In. aqua regia	Outcrop	34.74	0.4	0.07	0.579	0.02	0.007	1.57.E+01	2.E+00	5.E-01	2.3	0.3	0.07	0.57	0.02	0.007
PLG-C-66.35	Mudstone	15.38	113	In. aqua regia	Core	22.17	0.17	0.03	0.567	0.015	0.004	1.03.E+01	1.5.E+00	3.E-01	2.36	0.3	0.08	0.563	0.015	0.004
PLG-C-66.25	Mudstone	15.48	113	In. aqua regia	Core	33.92	0.3	0.05	0.403	0.008	0.003	1.07.E+01	3.E+00	6.E-01	1.58	0.4	0.09	0.400	0.008	0.003
PLG+6.5	Mudstone	15.5	113	In. aqua regia	Outcrop	55.4	1.0	0.15	0.516	0.016	0.005	9.7.E+00	2.E+00	5.E-01	0.9	0.2	0.05	0.514	0.016	0.005
PLG-C-66.10	Mudstone	15.63	113	In. aqua regia	Core	38.02	0.4	0.07	0.528	0.016	0.005	1.32.E+01	3.E+00	6.E-01	1.77	0.4	0.08	0.524	0.016	0.005
PLG+7.0	Marlstone	16	113	In. aqua regia	Outcrop	24.01	0.3	0.05	0.61	0.02	0.007	5.8.E+00	2.E+00	5.E-01	1.2	0.5	0.11	0.610	0.02	0.007
PLG+7.5	Marlstone	16.5	113	In. aqua regia	Outcrop	49.1	1.0	0.15	0.59	0.03	0.008	1.41.E+01	3.E+00	6.E-01	1.5	0.3	0.07	0.589	0.03	0.008
PLG-C-64.85	Marlstone	16.88	113	In. aqua regia	Core	62.9	0.7	0.10	0.706	0.013	0.004	1.660.E+03	8.E+01	1.7.E+01	136.7	6.5	1.4	0.449	0.018	0.005
PLG-C-64.85(2)	Marlstone	16.88	113	In. aqua regia	Core	58.3	0.2	0.03	0.737	0.005	0.002	1.636.E+03	8.E+01	1.8.E+01	146.0	7	1.6	0.462	0.015	0.003
PLG-C-64.85(3)	Marlstone	16.88	113	In. aqua regia	Core	57.0	0.4	0.06	0.746	0.011	0.003	1.631.E+03	6.E+01	1.5.E+01	148.9	6	1.3	0.465	0.016	0.004
PLG-C-64.70	Black shale (Kilian)	17.03	113	In. aqua regia	Core	233.3	2	0.3	1.506	0.02	0.008	2.42.E+04	4.E+02	1.E+02	590	12	3	0.395	0.03	0.009
PLG+8.1	Black shale (Kilian)	17.1	113	CrO_3-H_2SO_4	Outcrop	308	7	1.0	2.021	0.05	0.016	1.823.E+04	2.E+02	5.E+01	356	9	1.5	1.35	0.06	0.016
PLG-C-64.50	Black shale (Kilian)	17.23	113	In. aqua regia	Core	205.2	5	0.8	1.251	0.06	0.018	1.023.E+04	2.E+02	5.E+01	275	9	2	0.733	0.06	0.018
PLG-C-64.49	Black shale (Kilian)	17.24	113	In. aqua regia	Core	192	6	0.9	1.16	0.09	0.03	8.93.E+03	3.E+02	6.E+01	254	11	2	0.68	0.09	0.03
PLG-C-64.49	Black shale (Kilian)	17.24	113	In. aqua regia	Core	194.0	1.6	0.3	1.124	0.02	0.007	8.68.E+03	1.9.E+02	4.E+01	243.4	6	1.3	0.665	0.03	0.008
PLG-C-64.30	Marlstone	17.43	113	In. aqua regia	Core	33.7	0.3	0.04	0.585	0.008	0.003	7.71.E+01	4.E+00	9.E-01	11.65	0.6	0.13	0.563	0.008	0.003
PLG-C-64	Marlstone	17.73	113	In. aqua regia	Core	55.47	0.3	0.05	0.631	0.008	0.003	5.49.E+01	1.8.E+00	4.E-01	5.08	0.2	0.04	0.621	0.008	0.003
PLG+10.0	Marlstone	19	113	CrO_3-H_2SO_4	Outcrop	55.6	2	0.3	0.642	0.04	0.012	2.93.E+01	3.E+00	6.E-01	2.7	0.3	0.05	0.64	0.04	0.012
PLG+10.0	Marlstone	19	113	In. aqua regia	Outcrop	56.0	0.7	0.11	0.649	0.017	0.005	1.17.E+01	1.8.E+00	4.E-01	1.1	0.2	0.04	0.65	0.017	0.005
PLG-C-62.11-62.2	Black shale	19.575	113	In. aqua regia	Core	179.3	5	0.7	0.63	0.04	0.011	3.344.E+02	7.E+01	1.7.E+01	10	2	0.5	0.615	0.04	0.011
PLG-C-61.05-61.2	Mudstone	20.6025	112	In. aqua regia	Core	221.5	5	0.7	0.68	0.04	0.012	2.438.E+02	6.E+01	1.3.E+01	5.7	1.4	0.3	0.666	0.04	0.012
PLG+12.10	Mudstone	21.1	112	In. aqua regia	Outcrop	39.0	1.5	0.2	0.611	0.06	0.019	2.34.E+01	4.E+00	8.E-01	3.1	0.5	0.11	0.60	0.06	0.019
PLG-C-60-60.15	Mudstone	21.73	112	In. aqua regia	Core	31.80	0.3	0.05	0.562	0.012	0.004	1.54.E+01	1.3.E+00	3.E-01	2.47	0.2	0.05	0.557	0.012	0.004
PLG-C-58.86-58.87	Mudstone	22.865	112	In. aqua regia	Core	91.4	1.0	0.16	0.691	0.016	0.005	1.054.E+02	5.E+00	1.2.E+00	6.0	0.3	0.07	0.680	0.016	0.005
PLG-C-58.4-58.48	Mudstone	23.29	111	In. aqua regia	Core	31.98	0.3	0.04	0.668	0.017	0.005	1.557.E+02	7.E+00	1.5.E+00	25.1	1.1	0.2	0.622	0.017	0.005
PLG-C-57.97	Marlstone	23.76	111	In. aqua regia	Core	51.27	0.4	0.07	0.66	0.007	0.003	9.23.E+01	6.E+00	1.4.E+00	9.3	0.6	0.14	0.646	0.007	0.003
PLG+15.5	Marlstone	24.5	111	CrO_3-H_2SO_4	Outcrop	61.9	1.6	0.2	0.777	0.02	0.007	5.85.E+02	1.4.E+01	3.E+00	49	1.7	0.3	0.69	0.02	0.007
PLG+15.5	Marlstone	24.5	111	In. aqua regia	Outcrop	59.7	0.9	0.15	0.762	0.02	0.007	4.250.E+02	6.E+00	1.4.E+00	37.1	0.8	0.15	0.69	0.02	0.007
PLG-C-56.56-56.58	Black shale (Urbino)	25.16	111	In. aqua regia	Core	673	6	1.0	3.80	0.05	0.016	1.600.E+05	5.E+03	1.1.E+03	1693	54	12	0.67	0.11	0.03
PLG-C-56.56-56.58	Black shale (Urbino)	25.16	111	In. aqua regia	Core	673.6	4	0.6	3.728	0.04	0.013	1.591.E+05	3.E+03	7.E+02	1672	34	7	0.633	0.08	0.019
PLG-C-56.44-56.48	Black shale (Urbino)	25.27	111	In. aqua regia	Core	749	6	1.0	2.90	0.05	0.015	1.399.E+05	1.8.E+03	4.E+02	1225	19	4	0.635	0.06	0.017
PLG-C-56.44-56.48	Black shale (Urbino)	25.27	111	In. aqua regia	Core	729	10	1.5	2.884	0.06	0.019	1.347.E+05	1.5.E+03	3.E+02	1211	21	4	0.64	0.07	0.02
PLG+16.28	Black shale (Urbino)	25.28	111	CrO_3-H_2SO_4	Outcrop	744	6	1.0	3.716	0.05	0.014	8.40.E+04	1.4.E+03	3.E+02	798	15	3	2.24	0.06	0.016
PLG+16.28	Black shale (Urbino)	25.28	111	In. aqua regia	Outcrop	788	9	1.3	3.62	0.07	0.02	8.392.E+04	8.E+02	1.8.E+02	747	11	2	2.24	0.07	0.02
PLG-C-56.20	Marlstone	25.53	111	In. aqua regia	Core	131.2	0.8	0.12	1.374	0.013	0.004	9.23.E+03	1.5.E+03	3.E+02	394	65	14	0.65	0.12	0.03
PLG+17.0	Marlstone	26	111	CrO_3-H_2SO_4	Outcrop	65.9	0.6	0.09	1.081	0.02	0.006	9.62.E+02	4.E+01	8.E+00	79	3	0.7	0.93	0.02	0.006
PLG+17.0	Marlstone	26	111	In. aqua regia	Outcrop	67.9	1.7	0.3	1.035	0.04	0.012	9.33.E+02	2.E+01	5.E+00	74.0	3	0.5	0.90	0.04	0.012
PLG-C-54.95	Black shale (Leenhardt)	26.78	111	In. aqua regia	Core	73.5	2	0.4	0.629	0.04	0.012	1.187.E+02	8.E+01	1.7.E+01	8	5	1.2	0.614	0.04	0.012
PLG-C-54.50	Marlstone	27.23	111	In. aqua regia	Core	22.31	0.4	0.06	0.547	0.019	0.006	2.86.E+01	3.E+00	6.E-01	6.5	0.6	0.14	0.534	0.019	0.006
PLG-C-54.00	Marlstone	27.73	111	In. aqua regia	Core	54.32	0.6	0.09	0.666	0.02	0.006	9.87.E+01	3.E+00	7.E-01	9.4	0.3	0.07	0.649	0.02	0.006

Supplementary Table S5: Os isotopic analysis of the PLG section.

Sample Name	Lithology	Depth	Age	Digestion liquid	Os conc	1SD	1SE	187Os/188Os$_m$	1SD	1SE	Re conc	1SD	1SE	187Re/188Os	1SD	1SE	187Os/188Os$_i$	1SD	1SE
		(mbsf)	(Ma)		(pg g-1)						(pg g-1)								
463_58-2_44-50	Limestone	510.94	113	In. aqua regia	42.22	0.4	0.05	0.686	0.010	0.0015	28.8	1.3	0.3	3.52	0.17	0.04	0.679	0.010	0.0015
463_59-1_54-56	Limestone	519.04	113	In. aqua regia	42.29	0.3	0.04	0.777	0.008	0.0012	845	28	6.3	104.3	4	0.8	0.580	0.010	0.0019
463_59-1_68-71	Limestone	519.18	113	In. aqua regia	15.50	0.17	0.03	0.683	0.012	0.0019	161.2	3	0.6	53.72	1.1	0.2	0.581	0.013	0.0019
463_59-2_96-98	Marlstone	520.96	113	In. aqua regia	16.20	0.16	0.02	0.654	0.012	0.0018	23.1	3	0.7	7.35	1.0	0.2	0.641	0.013	0.0019
463_59-2_116-118	Limestone	521.16	113	In. aqua regia	12.38	0.12	0.018	0.657	0.013	0.0019	20.1	1.6	0.4	8.35	0.7	0.15	0.641	0.013	0.0019
463_59-3_133-135	Limestone	522.83	113	In. aqua regia	23.43	0.16	0.02	0.738	0.012	0.0018	46.2	3	0.6	10.26	0.6	0.14	0.719	0.012	0.0018
463_60-1-18_20	Limestone	528.18	113	In. aqua regia	14.94	0.3	0.05	0.612	0.03	0.005	2.3	1.2	0.3	0.80	0.4	0.09	0.611	0.035	0.005
463_60-1_44-46	Limestone	528.44	113	In. aqua regia	33.02	0.19	0.03	0.577	0.009	0.0014	19.0	1.2	0.3	2.94	0.19	0.04	0.572	0.009	0.0014
463_60-1_73-75	Limestone	528.73	113	In. aqua regia	18.50	0.3	0.04	0.597	0.018	0.003	6.4	2	0.5	1.75	0.6	0.14	0.593	0.018	0.003
463_60-1_100-102	Limestone	529	113	In. aqua regia	20.56	0.4	0.05	0.596	0.03	0.004	6.5	3	0.6	1.62	0.6	0.14	0.593	0.029	0.004
463_60-1_110-115	Limestone	529.1	113	In. aqua regia	17.18	0.13	0.019	0.596	0.011	0.0016	4.0	1.1	0.2	1.19	0.3	0.07	0.594	0.011	0.0016
463_60-1_122-127	Limestone	529.22	113	In. aqua regia	18.87	0.15	0.02	0.608	0.009	0.0014	5.7	1.1	0.2	1.55	0.3	0.07	0.605	0.009	0.0014
463_60-1_133-138	Marlstone	529.33	113	In. aqua regia	17.08	0.2	0.03	0.625	0.02	0.003	10.5	2	0.5	3.16	0.7	0.16	0.619	0.020	0.003
463_60-1_138-141①	Marlstone	529.38	113	In. aqua regia	26.00	0.2	0.03	0.582	0.010	0.0015	8.1	1.1	0.2	1.60	0.2	0.05	0.579	0.010	0.0015
463_60-1_138-141②	Marlstone	529.41	113	In. aqua regia	31.73	0.14	0.02	0.544	0.005	0.0008	16.6	1.2	0.3	2.66	0.2	0.04	0.539	0.005	0.0008
463_60-2_6-8	Limestone	529.56	113	In. aqua regia	33.67	0.12	0.019	0.540	0.004	0.0007	10.6	2	0.6	1.59	0.4	0.08	0.537	0.004	0.0007
363_60-2_27-29	Marlstone	529.77	113	In. aqua regia	37.93	0.3	0.05	0.575	0.010	0.0015	24.8	3	0.7	3.34	0.4	0.09	0.569	0.010	0.0015
463_60-2_46-49	Limestone	529.96	113	In. aqua regia	30.65	0.16	0.02	0.579	0.005	0.0007	15.0	1.2	0.3	2.50	0.2	0.05	0.575	0.005	0.0007
463_60-2_82-85	Marlstone	530.32	113	In. aqua regia	21.34	0.4	0.06	0.586	0.03	0.005	6.4	1.4	0.3	1.54	0.3	0.08	0.583	0.031	0.005
463_60-2_100-103	Limestone	530.5	113	In. aqua regia	20.26	0.2	0.03	0.559	0.013	0.002	7.0	3	0.6	1.77	0.7	0.15	0.556	0.013	0.002
463_60-3_8-12	Marlstone	531.08	113	In. aqua regia	49.64	0.4	0.06	0.438	0.006	0.0010	25.8	1.7	0.4	2.61	0.18	0.04	0.433	0.006	0.0010
463_60-3_36-40	Limestone	531.36	113	In. aqua regia	22.50	0.2	0.03	0.567	0.011	0.0016	9.7	1.3	0.3	2.19	0.3	0.06	0.563	0.011	0.0016
463_60-3_58-59	Limestone	531.58	113	In. aqua regia	30.47	0.18	0.03	0.545	0.007	0.0010	13.3	3	0.6	2.21	0.5	0.10	0.541	0.007	0.0010
463_60-3_111-117	Marlstone	532.11	113	In. aqua regia	42.15	0.4	0.05	0.547	0.012	0.0018	23.3	1.4	0.3	2.81	0.17	0.04	0.541	0.012	0.0018
463_60-3_130-134	Limestone	532.3	113	In. aqua regia	41.27	0.2	0.03	0.554	0.007	0.0010	20.8	1.3	0.3	2.57	0.16	0.03	0.549	0.007	0.0010
463_60-4_30-32	Limestone	532.8	113	In. aqua regia	25.06	0.3	0.04	0.510	0.013	0.002	8.9	1.1	0.2	1.80	0.2	0.05	0.506	0.013	0.002
463_60-4_52-54	Limestone	533.02	113	In. aqua regia	21.62	0.13	0.02	0.516	0.007	0.0010	4.6	0.9	0.2	1.07	0.2	0.05	0.514	0.007	0.0010
463_60-4_90-93	Limestone	533.4	113	In. aqua regia	34.03	0.2	0.03	0.530	0.009	0.0013	10.6	0.8	0.19	1.58	0.13	0.03	0.527	0.009	0.0013
463_60-4_110-117	Limestone	533.6	113	In. aqua regia	32.87	0.17	0.03	0.525	0.007	0.0010	13.3	0.8	0.18	2.05	0.13	0.03	0.521	0.007	0.0010
463_61-1_12-15	Limestone	535.62	114	In. aqua regia	28.03	0.11	0.016	0.539	0.006	0.0010	9.6	0.8	0.19	1.74	0.15	0.03	0.536	0.006	0.0010
463_61-1_47-49	Marlstone	535.97	114	In. aqua regia	21.54	0.18	0.03	0.528	0.010	0.0015	15.6	1.5	0.3	3.66	0.4	0.08	0.521	0.010	0.0015
463_61-1_103-105	Marlstone	536.53	114	In. aqua regia	27.65	0.19	0.03	0.519	0.008	0.0013	8.0	1.2	0.3	1.47	0.2	0.05	0.516	0.008	0.0013
463_62-1_3-5	Limestone	537.53	114	In. aqua regia	38.20	0.2	0.03	0.496	0.006	0.0009	86.7	1.9	0.4	11.45	0.3	0.06	0.474	0.006	0.0009
463_62-1_47-49	Marlstone	537.97	114	In. aqua regia	47.73	0.4	0.06	0.521	0.011	0.0017	50.1	1.8	0.4	5.32	0.2	0.04	0.511	0.011	0.0017
463_62-1_114-116	Marlstone	538.64	114	In. aqua regia	38.61	0.15	0.02	0.522	0.004	0.0006	65.4	2	0.5	8.57	0.3	0.07	0.506	0.004	0.0006
463_62-2_32-35	Limestone	539.32	114	In. aqua regia	52.41	0.4	0.06	0.514	0.011	0.0017	29.6	1.4	0.3	2.86	0.14	0.03	0.508	0.011	0.0017
463_62-2_80-81	Marlstone	539.8	114	In. aqua regia	31.66	0.11	0.016	0.516	0.005	0.0008	15.4	1.4	0.3	2.46	0.2	0.05	0.511	0.005	0.0008
463_62-2_113-115	Marlstone	540.13	114	In. aqua regia	35.13	0.18	0.03	0.501	0.005	0.0008	27.0	1.3	0.3	3.89	0.19	0.04	0.494	0.005	0.0008
463_62-3_83-88	Limestone	541.33	114	In. aqua regia	49.52	0.3	0.04	0.487	0.006	0.0010	23.8	1.1	0.2	2.42	0.11	0.03	0.483	0.006	0.0010
463_62-3_111-113	Marlstone	541.61	114	In. aqua regia	34.73	0.3	0.04	0.501	0.008	0.0012	24.3	1.3	0.3	3.54	0.2	0.04	0.494	0.008	0.0012

Supplementary Table S6: Os isotopic analysis of theDSDP Site 463

Sample Name	Section	Depth	Na2O	MgO	Al2O3	SiO2	P2O5	K2O	CaO	TiO2	Cr2O3	MnO	Fe2O3	NiO	LOI
		(m/mbsf)	wt%	wt%	wt%	wt%	wt%	wt%	wt%	wt%	wt%	wt%	wt%	wt%	
PLG-9	PLG	0 m	0.27	1.40	6.91	34.44	0.06	1.78	27.53	0.25	0.01	0.18	2.49	0.01	24.21
PLG-8.5	PLG	0.5 m	0.22	1.60	7.72	35.86	0.06	2.14	25.47	0.28	0.01	0.16	3.08	0.01	22.77
PLG-8	PLG	1 m	0.22	1.63	7.78	36.96	0.06	2.17	24.75	0.29	0.01	0.17	2.95	0.01	22.35
PLG-7.5	PLG	1.5 m	0.24	1.91	8.55	37.54	0.05	2.33	23.36	0.34	0.01	0.15	3.37	0.01	21.57
PLG-7	PLG	2 m	0.12	1.01	4.13	23.22	0.05	1.02	36.78	0.14	0.00	0.23	1.37	0.00	31.24
PLG-6	PLG	3 m	0.12	1.04	4.63	26.09	0.05	1.16	34.60	0.15	0.01	0.22	1.27	0.00	29.79
PLG-5	PLG	4 m	0.17	1.10	4.94	27.18	0.06	1.18	33.82	0.15	0.01	0.21	1.18	0.00	29.23
PLG-4	PLG	5 m	0.11	0.87	3.33	21.09	0.04	0.60	38.81	0.09	0.00	0.25	0.99	0.00	32.79
PLG-2	PLG	7 m	0.10	0.79	3.23	24.50	0.05	0.74	37.03	0.09	0.00	0.21	1.04	0.00	31.42
PLG0	PLG	9 m	0.13	1.09	5.12	32.44	0.09	1.36	30.46	0.15	0.01	0.16	1.97	0.00	26.45
PLG1	PLG	10 m	0.08	0.80	3.11	21.02	0.06	0.69	39.22	0.10	0.00	0.20	0.89	0.00	33.02
PLG2	PLG	11 m	0.16	0.97	4.64	28.57	0.07	0.95	33.35	0.13	0.00	0.16	1.72	0.00	28.58
PLG4	PLG	13 m	0.12	1.06	5.19	29.29	0.13	1.28	31.95	0.16	0.01	0.18	2.14	0.00	27.68
PLG4.5	PLG	13.5 m	0.16	1.09	5.60	30.84	0.10	1.31	30.98	0.16	0.01	0.16	2.32	0.00	26.85
PLG5	PLG	14 m	0.14	1.23	6.42	34.70	0.08	1.59	27.46	0.20	0.01	0.18	2.89	0.00	24.30
PLG6	PLG	15 m	0.18	1.31	6.97	41.68	0.09	1.66	23.29	0.21	0.01	0.17	2.81	0.00	21.09
PLG6.5	PLG	15.5 m	0.24	1.86	9.91	51.85	0.09	2.53	14.33	0.44	0.02	0.11	3.46	0.01	14.49
PLG7.5	PLG	16.5 m	0.15	0.96	5.36	41.90	0.06	1.08	25.45	0.13	0.01	0.21	1.38	0.00	22.56
PLG10	PLG	19 m	0.18	1.45	7.95	48.23	0.10	1.85	18.37	0.21	0.01	0.15	3.22	0.01	17.35
PLG15.5	PLG	24.5 m	0.19	1.24	7.63	46.07	0.09	1.57	20.97	0.23	0.01	0.13	1.90	0.01	19.19
PLG17	PLG	26 m	0.22	1.64	9.36	48.62	0.09	1.93	17.67	0.26	0.01	0.11	2.32	0.01	16.87
463_60-4_90-93	Site 463	533.4 mbsf	0.21	0.57	1.70	18.10	0.08	0.05	42.28	0.24	0.00	0.02	0.99	0.00	34.88
463_60-4_110-117	Site 463	533.6 mbsf	0.11	0.45	1.38	23.65	0.06	0.03	39.63	0.17	0.00	0.03	0.74	0.00	33.17
463_61-1_12-15	Site 463	535.62 mbsf	0.07	0.29	0.82	18.49	0.06	0.04	43.32	0.09	0.00	0.03	0.36	0.00	35.73
463_61-1_103-105	Site 463	526.53 mbsf	0.00	0.36	1.06	17.92	0.04	0.00	43.52	0.10	0.00	0.03	0.50	0.00	35.75
463_62-2_113-115	Site 463	540.13 mbsf	0.15	0.43	1.36	12.64	0.05	0.06	45.91	0.07	0.00	0.03	0.47	0.00	37.89
463_62-3_83-88	Site 463	541.33 mbsf	0.15	0.48	1.71	17.40	0.06	0.05	43.00	0.06	0.00	0.03	0.55	0.00	35.60
463_62-3_111-113	Site 463	541.61 mbsf	0.13	0.33	1.20	20.56	0.05	0.06	42.15	0.04	0.00	0.02	0.37	0.00	34.67

Supplementary Table S7: Major elemental composition of sedimentary rock samples of the PLG section and the DSDP Site 463.

Interval	Depth at PLG (msl)	Age interval (Ma)	R_{ocean}	M_{ocean} (t)	F_{cont} (t/ky)	F_{Kerg} (t/ky)	Remarks
Steady state	1~12.5	~5	0.54	1.51×10^4	295.0	0	Steady state
After jacob level	~13.5	~0.5	0.47	1.41×10^4	239.7	0	Decrease in continental weathering
			0.47	1.85×10^4	295.0	191.0	Hydrothermal activities at the SKP
Extinction horizon	15.48	<0.1	0.40	1.32×10^4	191.1	0	Decrease in continental weathering
			0.40	2.39×10^4	295.0	493.4	Hydrothermal activities at the SKP
Extinction horizon to Kilian level			0.58	1.57×10^4	330.2	0	Increase in continental weathering
Lower Kilian level	16.88-17.03	~0.6	0.40	1.32×10^4	191.1	0	Decrease in continental weathering
			0.40	2.75×10^4	330.2	660.5	Hydrothermal activities at the SKP
Upper Kilian level	~17.24	~0.6	0.73	1.86×10^4	493.2	0	Increase in continental weathering
Upper than Kilian level	17.24-	~2	0.62	1.64×10^4	368.5	0	Increase in continental weathering

Supplementary Table S8: Parameters and the results of Os-box model calculation.

Supplementary Notes

1. Geological settings and the stratigraphic information

1.1. Poggio le Guaine (PLG) Section

A representative Tethyan pelagic sedimentary sequence crops out at the Umbria–Marche basin of central Italy (Supplementary Fig. S1). Sedimentary rocks of the Poggio le Guaine (PLG) section is mainly composed of biogenic calcite (foraminifera and coccolith), biogenic silicate (radiolaria), and fine clay minerals and does not contain coarse sandy materials (Fig. 2a-c). The sedimentary sequences of this basin cover the Tithonian (Jurassic) to Lutetian (Paleocene) and can be subdivided into four formations in the ascending order: Maiolica (Tithonian to Early Aptian), Marne a Fucoidi (Early Aptian to latest Albian), Scaglia Bianca (latest Albian to earliest Turonian), and Scaglia Rossa (Early Turonian to Early Lutetian)[54]. The Upper Aptian to Lower Albian interval belongs to the Marne a Fucoidi Formation and are well-exposed at Poggio le Guaine (PLG) section, on the Monte Nerone ridge (Supplementary Fig. S1). This section once located at middle to lower bathyal depths of about 1000–1500 m. The paleolatitude was estimated to be about 20°N[4]. Detailed foraminiferal and carbon isotopic studies have revealed that PLG section covers Upper Aptian to Lower Albian and contains the OAE1b interval continuously[4].

1.2. Deep Sea Drilling Program (DSDP) Site 463

The DSDP Site 463 is located in the western Mid-Pacific Mountains and consists of 822.5 m-thick Cenozoic to Mesozoic pelagic carbonate sediments (Supplementary Fig. S2)[55]. The Upper Aptian to Lower Albian sedimentary interval was deposited at about 15°S (Supplementary Fig. S2)[55] DSDP Site 463 consists of vari-colored limestone with some chert layers. Bioturbation is common throughout the DSDP Site 463 cores 58-62 (Supplementary Fig. S5).

2. Stable carbon and oxygen isotopic composition of carbonate

The results of $\delta^{13}C_{carb}$ and $\delta^{18}O_{carb}$ analysis are listed in Supplementary Tables S1, S2. Considering the relatively linear correlation between $\delta^{13}C_{carb}$ and $\delta^{18}O_{carb}$ of PLG section (Supplementary Fig. S3a), these values might have experienced diagenetic alternation to some extent. Also, the fluctuation of $\delta^{13}C_{carb}$ is larger than that of the DSDP Site 463, which might be derived from diagenetic alternation. However, since the smoothed trend of $\delta^{13}C_{carb}$ at PLG section[4] is quite similar to that of other sections (e.g. Vocontian Basin in southeast France, DSDP Site 545 at Mazagan Plateau, and ODP Site

1049 at Blake Nose[2,5]), major trends of $\delta^{13}C_{carb}$ variation is considered to be preserved.

The $\delta^{13}C_{carb}$ and $\delta^{18}O_{carb}$ records of the DSDP Site 463 don't show a clear correlation, which implies that $\delta^{13}C_{carb}$ of this samples has not experienced the post-depositional alteration. Here, one sample (523.07 mbsf, core 59, section 4, 7 to 11cm) showing extremely low $\delta^{13}C_{carb}$ and $\delta^{18}O_{carb}$ values are excluded from the discussions as outliers (Supplementary Fig. S6b).

3. Micropaleontological analysis in DSDP Site 463

Our detailed analyses allowed the identification in the studied interval of three primary bioevents that are as follows in stratigraphic order: 1) the highest occurrence (HO) of *Paraticinella rohri* (= *Ticinella bejaouensis* and *Paraticinella eubejaouensis* in previous literature (see ref[4]) at 530.19 mbsf, 2) the lowest occurrence (LO) of *Microhedbergella miniglobularis* at 530.19 mbsf, 3) the LO of *Microhedbergella renilaevis* at 529.56 mbsf and 4) the LO of *Microhedbergella rischi* at 522.83 mbsf. *Paraticinella rohri* is already present at the base of the studied interval. Notably, an acme of the elongated chambered *Schackoina cepedai* is recorded at 539.8 mbsf and likely correlated with the similar interval recognized by ref[6] in the late Aptian at ODP Hole 1049C and by ref[4] at the Poggio le Guaine section. Our documentation demonstrates the reliability of this datum for global correlation. A major planktonic foraminiferal species turnover accompanied by a dramatic reduction in shell size, a fundamental change in shell architecture, and a marked drop in the abundance of planktonic species occurs near the Aptian/Albian boundary at 513.19 mbsf where the large-sized *P. rohri* and the co-occurring last long-ranging Aptian hedbergellids disappear. According to ref[56], the Aptian/Albian boundary is placed at 530.19 mbsf at the LO of *M. renilaevis*. Finally, the first specimens of *Ticinella primula* are found at 513.54 mbsf.

From all the above, the late Aptian-early Albian at DSDP Site 463 can be subdivided, from bottom to top, into the *P. rohri*, *M. miniglobularis*, *M. renilaevis*, *M. rischi* and *T. primula* Zones (Figs. 3, 4)

4. Re-Os isotopic measurements

4.1. PLG section

Re-Os data of PLG section is listed in Supplementary Fig. S8 and Table S5. Most of the present-day measured $^{187}Os/^{188}Os$ values ($^{187}Os/^{188}Os_m$) range between 0.4 and 0.7 (Supplementary Fig. S8). However, $^{187}Os/^{188}Os_m$ of Kilian and Urbino level showed higher values between 1.1 and 3.8, which reflects the effect of additional ^{187}Os decayed from ^{187}Re after deposition. Initial $^{187}Os/^{188}Os$ values ($^{187}Os/^{188}Os_i$) of most limestone,

marlstone, and shale samples range from 0.5 to 0.7 (Supplementary Fig. S8). However, in the PLG section, $^{187}Os/^{188}Os_i$ of organic-rich samples show a different trend between outcrop samples and borehole core samples. An outcrop sample of Kilian level and Urbino level shows particularly high $^{187}Os/^{188}Os_i$ values of 1.37 and 2.25 respectively which exceeds far beyond the possible range of Phanerozoic marine Os isotopic composition ($^{187}Os/^{188}Os$ ~0.2 to 1.5). On the other hand, PLG-core samples shows much lower values of 0.4 to 0.73 at the Kilian level and 0.63 to 0.66 at the Urbino level respectively. These features are evident in the cross plot of measured $^{187}Os/^{188}Os$-$^{187}Re/^{188}Os$ (Supplementary Fig. S12). All PLG-core samples are scattered around the isochrone of 114 Ma. However, black shale samples collected from the outcrop are apparently out of this trend. Those unusually higher $^{187}Os/^{188}Os_i$ values in the PLG outcrop could reflect the post-depositional alternation caused by weathering. Ref[57,58] showed that weathering of organic- and Re-rich samples could change their $^{187}Os/^{188}Os_i$ values because of the different post-depositional mobilities between Os and Re. This time, large Re loss or addition of Os occurred during diagenesis or weathering. This effect is crucial to the samples with high Re concentration which require large age correction. In the PLG section, the sedimentary rocks upper than the extinction interval of planktonic foraminifera are fragile and TOC and Re concentration are higher than pre-OAE1b intervals. We concluded that fragile black shale, marlstone, and mudstone samples with high TOC and Re/Os ratio are susceptible to weathering. Hereafter, we exclude four outcrop samples with high-Re concentrations at 15.5 msl, 17.1 msl, 25.28 msl, and 26 msl from the further discussions (Supplementary Fig. S12). Only limestone, mudstone, and marlstone samples with low-Re concentrations whose differences of measured and initial $^{187}Os/^{188}Os$ are less than 0.01 are considered here.

4.2. DSDP Site 463

Os and Re concentration and isotopic ratio data are listed in Supplementary Fig. S9 and Table S6. Age correction does not affect $^{187}Os/^{188}Os_i$ too much because of the low Re concentration throughout this core. Therefore, $^{187}Os/^{188}Os_m$ and $^{187}Os/^{188}Os_i$ show similar variation.

4.3 Rhenium and osmium records across the Aptian/Albian boundary

A study by ref[41] has proposed that digestion with CrO_3-H_2SO_4 properly extracts Re and Os in hydrogenous fraction of sediments, while digestion with inverse aqua regia dissolved Re and Os in terrigenous fraction of sediments. Here we tested both digestion methods for some samples of PLG section and found no significant differences in the

initial $^{187}Os/^{188}Os$ values ($^{187}Os/^{188}Os_i$) (Supplementary Figs. S8, S13). This correspondence of $^{187}Os/^{188}Os$ values between these methods suggests that Os derived from the terrigenous material is minor in these samples and inverse aqua regia adequately extracts Re and Os in the hydrogenous fractions of sediments. However, Re concentrations dissolved by the inverse aqua regia are a little lower than that dissolved by CrO_3–H_2SO_4 (Supplementary Figs. S8, S13c). Since the aqua regia dissolves not only hydrogenous fraction but also silicate minerals and extract more Re from sediments, the cause of these differences is corundum. However, these differences are too small to affect $^{187}Os/^{188}Os_i$.

The trends and most of the $^{187}Os/^{188}Os_i$ of the two distant sites (PLG and DSDP Site 463) are quite similar. This similarity also suggests that our $^{187}Os/^{188}Os_i$ records reflect hydrogenous Os information strongly though there might be slight contamination of silicate materials.

References for supplementary information

53. Cresta, S., Monechi, S. & Parisi, G. *Mesozoic–Cenozoic stratigraphy in the Umbria–Marche Area. Geological field trips in the Umbria–Marche Apennines (Italy), Mem. Descr. Carta Geol. Ital.* 39, 48-49 (1989).
54. Coccioni, R. & Galeotti, S. The mid-Cenomanian Event: prelude to OAE 2. *Palaeogeography, Palaeoclimatology, Palaeoecology* **190**, 427-440 (2003).
55. Thiede, J. *et al*. Western Mid-Pacific Mountains. *In* Thiede, J., et al., *Initial Reports of Deep Sea Drilling Project, vol. 62*: Washington (U.S. Government. Printing Office), 33-156 (1981).
56. Kennedy, J. W. *et al*. The global boundary stratotype section and point (GSSP) for the base of the Albian stage, of the Cretaceous, the Col de Pré-Guittard section, Arnayon, Drôme, France. *Episodes* **40.3** 177-188 (2017).
57. Peucker-Ehrenbrink, B. & Hannigan, R. E. Effects of black shale weathering on the mobility of rhenium and platinum group elements. *Geology* **28**, 475-478 (2000).
58. Georgiev, S. *et al*. Chemical signals for oxidative weathering predict Re–Os isochroneity in black shales, East Greenland. *Chemical Geology* **324**, 108-121 (2012).
